# Piezoelectric nanofiber–based intelligent hearing system

**DOI:** 10.1126/sciadv.adl2741

**Published:** 2025-05-07

**Authors:** Jinke Chang, Thomas Maltby, Amirbahador Moineddini, Daqian Shi, Lei Wu, Jishizhan Chen, Jianshu Yu, Jeffrey Hung, Giuseppe Viola, Antonio Vilches, Wenhui Song

**Affiliations:** ^1^ UCL Centre for Biomaterials in Surgical Reconstruction and Regeneration, Department of Surgical Biotechnology, Division of Surgery & Interventional Science, University College London, London NW3 2PF, UK.; ^2^ The UCL Institute of Health Informatics, University College London, 222 Euston Rd., London NW1 2DA, UK.

## Abstract

Hearing loss, affecting individuals of all ages, can impair education, social function, and quality of life. Current treatments, such as hearing aids and implants, aim to mitigate these effects but often fall short in addressing the critical issue of accurately pinpointing sound sources. We report an intelligent hearing system inspired by the human auditory system: asymmetric well-aligned piezoelectric nanofibers combined with neural networks to mimic natural auditory processes. Piezoelectric nanofibers with spirally varying lengths and directions transmit and convert acoustic sound into mechanoelectrical signals, mimicking the complex cochlear dynamics. These signals are then encoded by digital neural networks, enabling accurate sound direction recognition. This intelligent hearing system surpasses human directional hearing, accurately recognizing sound directions horizontally and vertically. The advancement represents a substantial stride toward next-generation artificial hearing, harmonizing transduction and perception with a nature-inspired design. It promises for applications in hearing aids, wearable devices, and implants, offering enhanced auditory experiences for those with hearing impairments.

## Teaser

A nature-inspired intelligent hearing system based on piezoelectric nanofibers achieves precise sound direction recognition.

## INTRODUCTION

Hearing is a complex process involving the reception of acoustic waves and their perception in the brain (*1*). It is one of the most important senses for animals to localize predators and capture food in nature and for humans to communicate or enjoy music. The auditory system’s role in this process is crucial. In mammals, the “cochlea” in the ear transduces the acoustic signal into electricity through the unique coiled structure of the basilar membrane and hair cells sitting on top of the basilar membrane (*2*). Inner hair cells collect and relay sound information to the brain through the auditory nerves (*3*, *4*).

Sound recognition and localization are essential for human-environment interactions, intricately governed by the auditory system. Acoustic signals are analyzed through intensive (*5*), spectral (*6*), temporal (*7*), and spatial (*8*, *9*) cues. In humans, binaural hearing relies on comparing interaural time differences (ITDs) (*10*, *11*) and interaural level differences (ILDs) (*12*), resulting from variations in sound wave arrival times and intensities at each ear [known as the “duplex” theory (*13*)]. Brain processing generates an auditory map for horizontal sound source determination, while vertical localization proves more complex because of limited ITD and ILD cues. Vertical cues, such as spectral filtering (*14*) and individual-specific head-related transfer functions (*15*, *16*), are uniquely weight averaged for sound localization (*17*). Head-related transfer functions modify sound spectra based on the arrival direction [known as unique transfer function (*18*)], enabling accurate sound positioning. The combination of auditory and visual cues (*19*) facilitates three-dimensional spatial mapping. Replicating these mechanisms through artificial means is pivotal for treating hearing disorders (*20*).

Inspired by the intricate human auditory system, artificial hearing devices are designed to mimic its functions, bolstering auditory capabilities for individuals with impairments. These advancements, including cochlear and brainstem implants, replace the function of damaged ear components by directly stimulating the auditory nerve or brainstem (*21*, *22*). These devices are built through multistage processing, mirroring the human auditory system’s architecture. They encompass a “cochlea-like” stage where sound is transduced into electrical signals via microphones and electrode arrays, effectively emulating the natural cochlear function. Following a trajectory resembling the natural auditory system, this process involves an “auditory midbrain” responsible for signal acquisition and preprocessing, an “auditory cortex” for feature extraction, and a trainable neural network for generating brain responses (*23*, *24*). However, individuals with these auditory implants still face spatial sound localization challenges because of inadequate spatial cues (*25*, *26*). While bilateral cochlear implants enhance sound direction perception by providing binaural cues for the brain to analyze ITDs and ILDs (*27*), challenges persist in vertical localization because of subtle ITD and ILD cues in the vertical plane (*28*), in interpreting monaural cues for the sound source direction (*29*), and in accurately estimating distances influenced by complex environmental factors (*30*), as well as multiple long-term complications after cochlear implantation (*31*). The brainstem implant bypasses the cochlear stage, relying on midbrain and auditory cortex signal processing to generate mappings for the sound direction, thus remaining constrained in its spatial capabilities.

Biomimetic and bioinspired acoustic systems have garnered substantial interest, offering an advanced route to emulate the auditory process (*32*). Piezoelectric acoustic devices present a highly promising avenue, directly converting acoustic waves into electric signals at designed resonance frequencies, mirroring the cochlea’s basilar membrane functions (*33*–*35*). Multiresonant piezoelectric acoustic devices, imitating basilar membrane structural frequency selectivity, have demonstrated heightened sensitivity and wider frequency coverage (*36*). The natural basilar membrane in the human cochlea has graded mass and stiffness along its length, exhibiting anisotropic mechanics and enabling nonlinear mechanosensory cochlear responses (*37*), encompassing broad frequency selectivity and auditory neuron reactions for further processing in the auditory system (*38*, *39*). However, current cochlear implants, for instance, have a limited range of channel (<24 channels of frequency) capacities, through which sound frequencies can be compared to the basilar membrane with ~3500 channels, thus far beyond the ideal treatment for the entire spectrum of hearing impairment. As a result, patients have limited perception of music, semantic tones, and speech in background noise. Moreover, multiresonant piezoelectric devices, tuned to first-order resonant frequency (*40*, *41*), often disregard multiorder resonance signals containing full spectral cues because of complexity. This, coupled with recognition algorithms relying solely on first-order responses, results in incomplete sound cues and inaccurate recognition, particularly for vertical sound localization. Integrating these nonlinear aspects into the front end of machine hearing systems yields notable performance enhancements (*42*, *43*). Recent advancements in deep learning and neural networks notably amplify the efficiency of artificial hearing systems by analyzing intricate sound data (*44*), aspiring to emulate human hearing and capture featured sound propagation cues (*45*–*48*).

**Fig. 1.**
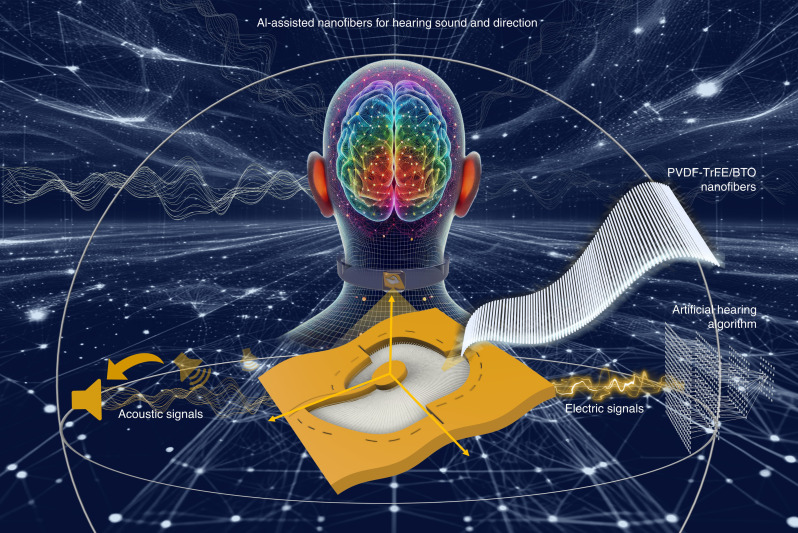
Schematic picture of an AI-assisted piezoelectric nanofiber–based spiral trampoline–like acoustic device for hearing sound and direction. Illustration of the radially aligned piezoelectric nanofibers (PVDF-TrFE/BTO) with tunable lengths transmitting and converting acoustic signals for hearing sound and direction with the assistance of AI.

We present an artificial hearing system integrating a piezoelectric nanofiber–based acoustic device and machine learning to replicate the auditory process. Physical sensing and transduction of sound signals are achieved by radially aligned piezoelectric nanofibers with gradually changing length and orientation, forming a spiral trampoline–like structure. Nanofiber resonant gradients emulate ILD-like signals containing intensity level differences via frequency selective vibration. Channel differences capture ITD-like signals containing time-dependent differences, while asymmetric geometry filters the directional spectrum of acoustic signals. Machine learning–based digital intelligence further identifies essential features from these piezoacoustic signals, enabling precise sound direction recognition (Fig. 1). The nanofiber-based artificial hearing system demonstrates a promising artificial model for mimicking the acoustic transduction and perception in an auditory system and paves the way to the next generation of artificial hearing systems.

## RESULTS

### Design of the trampoline-like multiresonant piezoacoustic devices with radially aligned nanofibers

**Fig. 2.**
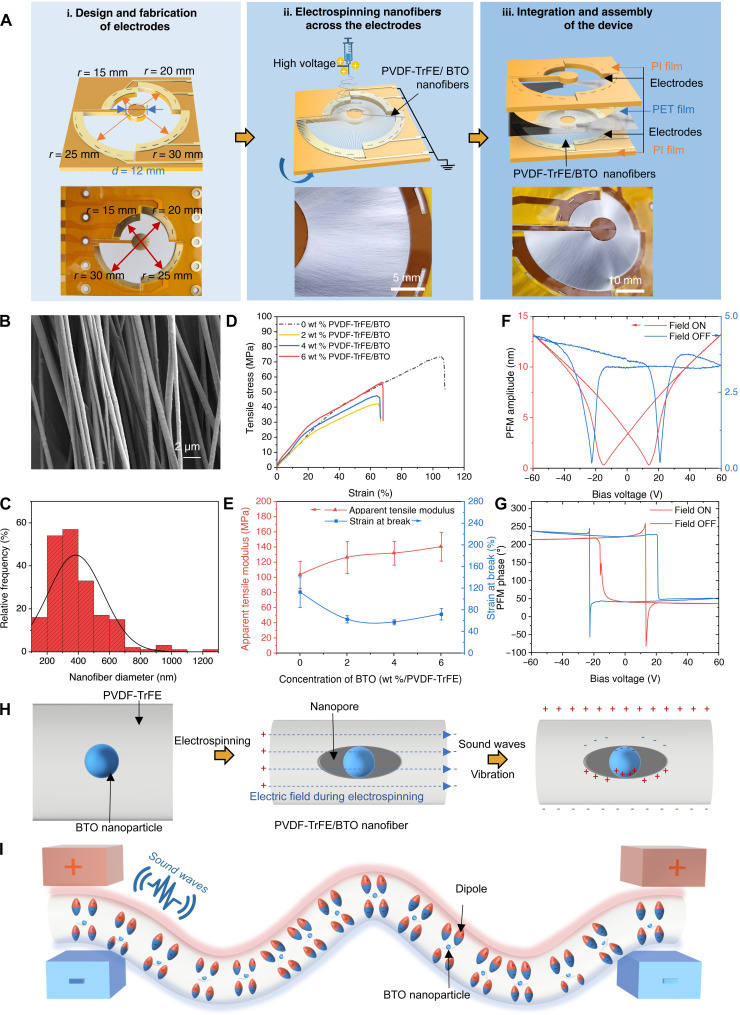
Characterization of PVDF-TrFE/BTO nanofibers. (**A**) Schematic illustration of design and fabrication of the spiral trampoline–like device: (i) design and fabrication of electrodes with step-changing channel diameters (30, 40, 50, and 60 mm); (ii) electrospinning of radially aligned nanofibers on the spiral electrodes by electrospinning; (iii) integration of the spiral trampoline–like device. The ratio of the contact surface areas of the two electrodes is kept constant by varying the diameter of the outer circle and the area of the inner electrode. (**B**) Field emission scanning electron microscopy micrograph of the nanofibers. (**C**) Histogram plot of the diameter of well-aligned PVDF-TrFE/BTO (6 wt %) nanofibers between two electrodes. (**D**) Strain/stress and (**E**) apparent tensile modulus and break point of electrospun fibers with different concentrations of BTO NPs (0, 2, 4, and 6 wt %). (**F**) PFM amplitude and (**G**) PFM phase of PVDF-TrFE/BTO (6 wt %) fiber with ±60-V voltage bias applied on a point in the polymer part. (**H**) Schematic illustration of the symbolic acoustic-piezoelectric principle at PVDF-TrFE/BTO NP nanocomposite interfaces and (**I**) piezoacoustic voltage potential of the nanofibers between two electrodes of the ST-PiezoAD device.

Inspired by human cochlea, a spiral trampoline–like piezoelectric acoustic device (ST-PiezoAD) is designed and made of outer and inner electrodes. To study the complexity of the piezoacoustic multiresonant responses of nanofibers with varying radii between continuous spiral-shaped electrodes, the electrodes were initially simplified by partitioning into four channels with a variable of radial in a millimeter step change. The outer electrodes are split into multiple electrodes, each of which spans a quarter of the external circular contour with a different radius, 15, 20, 25, and 30 mm, as shown in Fig. 2A(i). The inner circular electrode is placed at the center, connected to the external contour in a cantilever-type manner. The macrosize devices are easy to fabricate, assemble, and characterize for the early stage of proof of concept and suitable for the application of wearable devices and serve as an interface for in vitro study on auditory neuron cells’ response. In principle, the size of the device can be scalable, either upscaling or downscaling, depending on the performance and application required. The number of outer electrode channels can be increased as desired from the macro- to nanoscale, ultimately accommodating single nanofibers per channel.

The spiral pairs of electrodes enable to assemble radially aligned piezoelectric nanofibers through electrospinning of poly(vinylidene fluoride-*co*-trifluoroethylene) copolymer/barium titanate nanoparticle (PVDF-TrFE/BTO NP) nanocomposite solution (Fig. 2, A and B). During the electrospinning process, the inner and outer electrodes were grounded to create a local electric field, facilitating the radial alignment of the piezoelectric nanofibers with varying length across electrodes to achieve a multiresonance response, resembling the natural basilar membrane of the cochlea. The fibers exhibited a perpendicular orientation to the electrodes and a parallel arrangement to each other [Fig. 2A(ii)]. Following electrospinning, an upper electrode and a polyethylene terephthalate (PET) film were assembled with the PVDF-TrFE/BTO nanofiber–covered bottom electrode as a sandwich-type structure, forming an ST-PiezoAD device [Fig. 2A(iii)]. In response to sound stimulation, the radially aligned piezoelectric nanofibers in the PiezoAD devices vibrate as springs and transmit sound waves across the spiral channel, generating spectra of resonant vibrations and voltage potentials (piezoacoustic signals) depending on the dimensions and shape of the spiral channel.

### Characterization of the piezoelectric composite nanofibers

The PVDF-TrFE/BTO nanofibers were thoroughly characterized to gain a comprehensive understanding of the various factors influencing their vibrational and sensing behavior. The optimal concentration of BTO NPs was determined to be 6 wt % relative to the weight of PVDF-TrFE. The morphology of the nanofibers was examined using scanning electron microscopy and scanning transmission electron microscopy, as depicted in Fig. 2B and fig. S1 (A and B). These images revealed a homogeneous distribution of BTO NPs within the PVDF-TrFE polymer matrix. Wide-angle x-ray scattering (WAXS) and small-angle x-ray scattering (SAXS) patterns in fig. S1 (E to F) reveal the crystalline structure and orientation, lamellae distribution, and fibril alignment in the PVDF-TrFE/BTO nanofibers.

The mechanical properties of the nanofibers were influenced by the amount of BTO NPs. Tensile tests were conducted on the electrospun fibers with varying concentrations of BTO NPs: 0, 2, 4, and 6 wt %. The strain-stress plots exhibited ductile characteristics with a high ultimate strain. The addition of rigid BTO NPs to the flexible polymer matrix resulted in an increase in the apparent modulus of the nanofibers but compromised by lower strength and ultimate strain because of the interfaces between the polymer and BTO NPs. The apparent modulus of the pure PVDF-TrFE nanofiber mat is measured as 103.6 ± 17.8 MPa and increased to 140.6 ± 18.9 MPa by adding 6 wt % BTO NPs. The maximum strain of pure PVDF-TrFE nanofiber mat is 113.1 ± 28.1% but decreased to 62.9 ± 6.6% by adding 6 wt % BTO NPs (Fig. 2, D and E). Atomic force microscopy (AFM) was used to study the nanomechanics of individual nanofibers (fig. S1, C and D). The surface modulus of the composite nanofibers exhibited a noticeable contrast between the NP and polymer areas, ranging from ~8 to 15 MPa (fig. S1D).

A distinctive arc-shaped diffraction pattern perpendicular to the nanofiber’s long axis in two-dimensional (2D) WAXS (fig. S1, E and G) corresponds to the β-phase (110/200) crystal structure of PVDF-TrFE (*q* = 1.41 Å^−1^, 2θ = 19.9°), indicating dominant β-phase crystals aligned along the nanofiber direction. A discernible shoulder at *q* = 1.26 Å^−1^ (2θ = 17.9°) indicates the presence of the α phase in a lower amount. The crystalline orientation rate of 79.77% was estimated from the azimuthal angle plot integrated at *q* = 1.41 Å^−1^. The crystallinity degree of the β phase was estimated as 51.6% from the 1D WAXS pattern of pure PVDF-TrFE nanofibers, while by adding BTO NPs, the crystallinity increased to 83.5%. An outer isotropic ring corresponding to sharp peaks located at 2θ = 31.5° (*q* = 2.2 Å^−1^) identifying the (110) plane of BTO NPs indicates the randomly oriented BTO nanocrystals (fig. S1E). Correspondingly, elliptical shape scattering of the 2D SAXS pattern (fig. S1, F and H) suggests the existence of a lamellar structure with a certain orientation.

The nanofibers were getting poled during the electrospinning process. Piezoelectric force microscopy (PFM) provides evidence of the ferroelectric nature of the nanofibers. The bias voltage-amplitude loops exhibited a characteristic butterfly-like shape, with minimum points observed at ~±15 and ±21 V for the on-field and off-field conditions, respectively (Fig. 2F). Phase shifts of 180° were observed in both the on-field and off-field loops (Fig. 2G), indicating the induced reorientation of polarization at the corresponding electric bias voltages. Further analysis of the local piezoelectric effect at the nanoscale measured by PFM indicates that the addition of BTO NPs has notably enhanced piezoelectricity of composite nanofibers compared to pure PVDF-TrFE nanofibers (table S2). To our best knowledge, the measured coefficient using PFM only represents the piezoelectric responses in nanoscale area under a specific condition. Therefore, it might not be comparable with *d*_33_ values of the bulk sheets measured by conventional quasistatic methods and conditions in the literature. Regardless the limitations, the enhanced voltage output of the PVDF-TrFE/BTO nanofiber–based device in the following section also confirmed the contribution of BTO NPs in the composite fiber, in agreement with the high piezoelectric performance of PVDF-TrFE/BTO composites and their device reported in the literature (table S2).

Despite the increase in the composite nanofiber’s piezoelectricity, the contribution of the high piezoelectricity of BTO NPs is not as high as expected because of PVDF-TrFE polymer wrapping and their multidomain structure and limited poling condition. It has been suggested that the interface between BTO NPs and the PVDF-TrFE matrix plays a more important role in the improvement of the piezoelectric effect of the composites (*35*). A nanointerface or pores around the BTO NPs along the well-aligned PVDF-TrFE matrix could be generated under an electric field during electrospinning (Fig. 2H). Upon acoustic vibrations, the local forces at the interfaces are bound to induce additional electric dipoles between the interfaces (*49*). The electric potential is then built up along the nanofiber and collected at the two ends of electrodes, which carry both rich information of fiber vibration and sound signal stimulation (Fig. 2I).

### Multiresonant behavior and tunable sensitivity of the trampoline-like PiezoADs

**Fig. 3.**
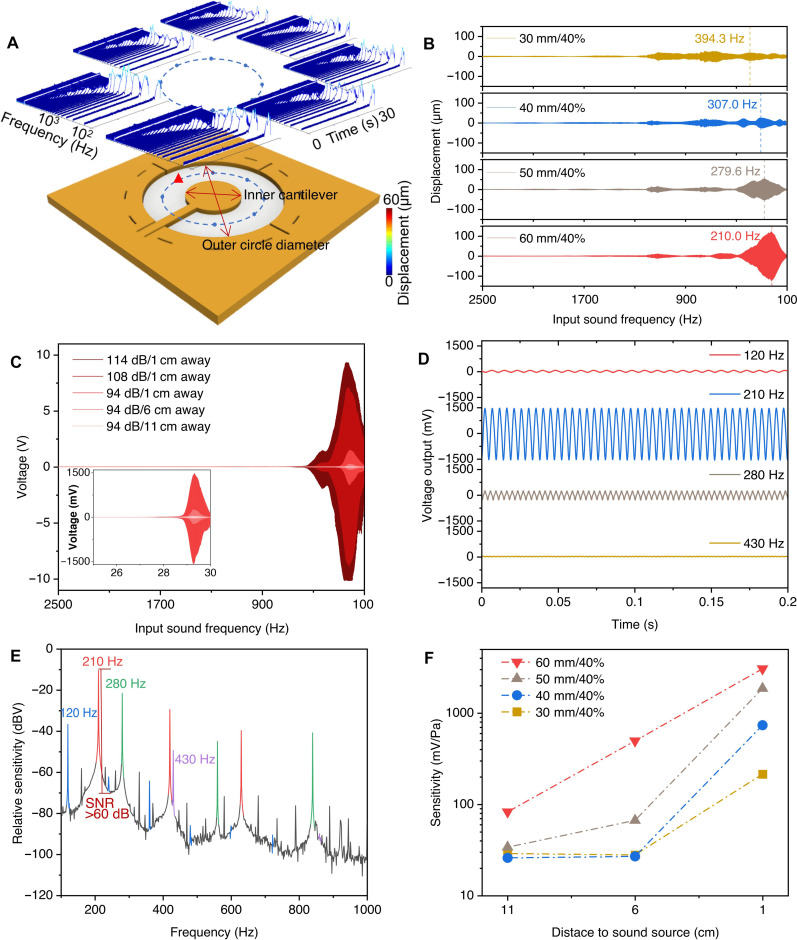
Tuning the performance of CT-PiezoADs. (**A**) STFT of the displacements of the symmetric points for a 30-mm/40% device, stimulated by a sweep sound frequency of 2500 to 100 Hz (the inner cantilever proportion is defined as the ratio of the inner diameter and the outer diameter). (**B**) Displacement for devices with a 40% cantilever proportion and outer diameters of 30, 40, 50, and 60 cm, measured from the red point highlighted in (A), under an SPL of 94 dB. (**C**) Voltage outputs of the 60-mm/40% device with SPLs of 94, 108, and 114 dB and sound sweep frequency from 2500 to100 Hz; inset: SPL of 94 dB and speaker distances to the device of 1, 6, and 11 cm. (**D**) Voltage outputs in response to monochromatic sound waves with frequencies of 120, 210, 280, and 430 Hz, with an SPL of 94 dB and a 1-cm distance from the speaker. (**E**) Sensitivity to monochromatic sound waves with different frequencies, converted from (D) (the fundamental frequencies are displayed in different colors, while the peaks of their higher-order harmonics are shown in the same colors). (**F**) Maximum sensitivity for the devices with a 40% cantilever proportion and outer diameters of 30, 40, 50, and 60 mm, with an SPL of 94 dB and sound sweep frequency in the range of 2500 to 100 Hz.

The vibration and voltage output of the ST-PiezoAD device are com-plex. To understand the impact of device geometry on multiresonant behavior and voltage outputs, we started to design and investigate a series of simplified circular trampoline–like piezoacoustic devices (CT-PiezoADs; Fig. 3) with varying outer circle diameters (30, 40, 50, and 60 mm) and inner circle area proportions (40, 60, and 80%) (fig. S2A). The inner cantilever proportion is defined as the ratio of the inner diameter to the outer diameter, which affects the area proportions or the width of nanofibers between two electrodes. Like ST-PiezoAD, the electrodes were designed with constant areas regardless of changes in the device’s geometry, ensuring a consistent contact area between the piezoelectric nanofibers and the electrodes. PVDF-TrFE/BTO (6 wt %) nanofibers were electrospun onto the electrodes and integrated as a sandwiched CT-PiezoAD. The uniform nanofiber alignment was achieved through a radially distributed electric field, with a maximum strength of 2.5 × 10^4^ V/m, as analyzed by COMSOL simulations (fig. S2B).

The devices’ resonant behavior was analyzed by monitoring the displacement of representative center points in each channel (middle points of the fiber length) in response to a sweep of sound frequencies from 5 kHz to 100 Hz in 30 seconds. Short-time Fourier transform (STFT) was performed to evaluate the frequency spectrum of the CT-PiezoADs. Figure 3A illustrates scans conducted on the 30-mm/40% device (outer circular diameter of 30 mm/inner cantilever proportion of 40%). The displacements of seven representative points around the dashed line were monitored, and the responsive spectrum was plotted in 3D, displaying the vibrational displacement in both time and frequency domains. Displacement peaks appeared at similar frequencies for symmetric points on both sides of the cantilever, indicating similar vibration behavior and resonance frequencies because of the device’s symmetry. The resonant behavior of CT-PiezoADs is adjusted by altering the spring length of the nanofibers. Figure 3B displays the representative displacement of CT-PiezoADs with different diameters. The first-order resonance frequency downshifted ranging from 394.3 to 210.0 Hz, and the largest displacement increased as the outer diameter increased from 30 to 60 mm, maintaining the same inner cantilever proportion of 40%. Figure S3A demonstrates changes in the first-order resonant frequency with varying inner cantilever proportions. The first-order resonant frequency increased from 394.3 to 548.0 Hz as the inner cantilever proportion varied from 40% to 60 and 80%, with the same outer diameter of 30 mm. As expected, the vibrational displacement increased with the length of the nanofiber springs.

Figure 3C illustrates a typical voltage output generated by a 60-mm/40% device under acoustic stimulation. As the sound pressure level (SPL) increased up to 114 dB, the maximum peak-to-peak voltage (*V*_*PP*_) of the device reached 19.41 V when resonating at ~210 Hz. The voltage output decreased as the distance from the device increased because of increased dispersion of sound energy. The CT-PiezoAD also exhibited multiple orders of resonant frequencies. Using the 60-mm/40% device as an example, we observed multiple peaks at 120, 210, 280, and 430 Hz, respectively (Fig. 3, B and D). We proceeded to characterize the responsive voltage outputs activated by monochromatic sound waves at these identified resonant frequencies. The maximum *V*_*PP*_ of 3022 mV was detected at 210 Hz with an SPL of 94 dB (Fig. 3D). The relative sensitivity (in dBV units) to different monochromatic sound waves of the 60-mm/40% device is calculated using Eq. 1 (*36*)

$$\text{Relative Sensitivity}\left(\text{dBV}\right)=20\text{log}\frac{V}{V_{0}}$$ (1)

where *V* is the root mean square value of the voltage output, and *V*_0_ is the reference of 1 V defined as 0 dBV. The relative sensitivity is plotted as a function of the sound frequency in Fig. 3E. A maximum relative sensitivity of −10.4 dBV is observed without an amplifier from the first resonance frequency of the 60-mm/40% device at 210 Hz. The device also demonstrated an excellent signal-to-noise ratio of >60 dB.

The spring effect of the aligned piezoelectric nanofibers plays an important role in the performance of CT-PiezoADs. The complete dataset of acoustic responsive voltage output for CT-PiezoADs with different diameters and cantilever proportions can be found in fig. S3(i). The maximum *V*_PP_ values for the 30-, 40-, and 50-mm devices (with a cantilever proportion of 40%) are also noteworthy, measuring 16.13, 19.37, and 18.17 V, respectively, under an SPL of 114 dB (fig. S3, C to H). The cantilever proportion also significantly changes the displacements and voltage outputs, as demonstrated by experiments conducted on the 30-mm device with cantilever electrode proportions of 40, 60, and 80% (fig. S3, A and B). Figure 3F summarizes the size effect on the sensitivity of the CT-PiezoADs with the optimized disc cantilever electrode (40%), converted from the peak voltages with Eq. 2 (*50*, *51*)

$$\text{Sensitivity (mvV/Pa)}=\frac{V}{P}=\frac{V}{P_{0}\times 10^{\frac{L_{p}}{20}}}$$ (2)

where *P*_0_ is the reference sound pressure of 0.00002 Pa, *L*_*p*_ is the SPL in decibel units, and *V* is the maximum peak voltages. The 60-mm/40% device showed a maximum sensitivity of 1532.9 mV/Pa. CT-PiezoADs with a larger outer diameter show higher sensitivity owing to the larger displacement generated by longer nanofiber springs.

**Fig. 4.**
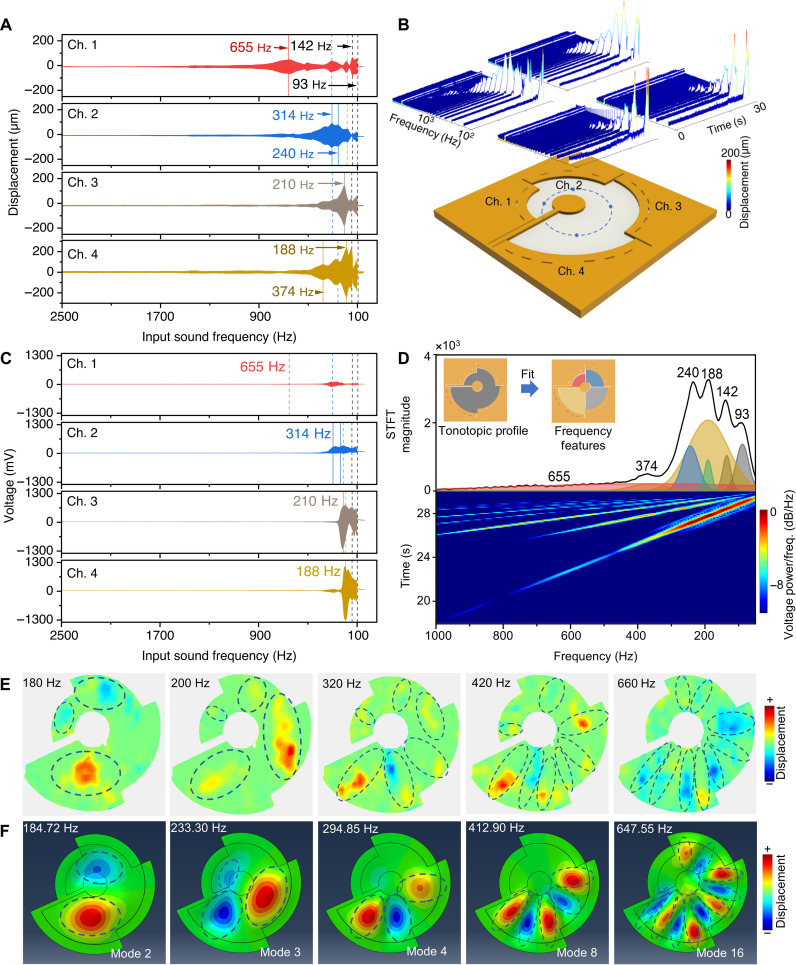
Piezoacoustic performance of the ST-PiezoAD. (**A**) Displacements and (**B**) STFT spectrum of the representative points of channels 1 to 4, measured from the blue dots highlighted in (B). (**C**) Voltage output of channels 1 to 4. The device was activated using a sound sweep spanning a frequency range from 2500 to 100 Hz. (**D**) Tonotopic profile derived from the voltage output of the ST-PiezoAD with four channels connected in series for an overall output. (**E**) Experimental mapping of the vibrational displacement of the ST-PiezoAD. (**F**) Vibrational mode simulation of the ST-PiezoAD.

The ST-PiezoAD features four channels with nanofibers in varying lengths following the same analytical approach. The vibration behavior, voltage transduction, and tonotopic profile of the ST-PiezoAD are systematically evaluated. Figure 4 (A and C) presents the vibration displacements and voltage outputs of each channel, respectively. The blue dots highlighted in Fig. 4B represent the monitored vibration displacement of each channel’s representative center point. The resonant behavior of each channel in the ST-PiezoAD is distinct. Channel 1 exhibits a resonant frequency of 655 Hz, while channels 2, 3, and 4 resonate at frequencies of 310, 210, and 190 Hz, respectively.

Piezoacoustic signal analysis suggests that the voltage output of each channel is influenced by its neighboring channels. For example, the resonant frequency of channel 4 at 190 Hz overlaps with a shoulder peak in channel 3, whose resonant frequency is at 210 Hz and is also reflected in channel 2. In addition, a resonance peak at 120 Hz is observed in both displacements and voltage outputs across all channels, which can be attributed to the resonant vibration of the inner cantilever. The STFT of the displacement at the representative points, stimulated by a sound frequency sweep, reveals the time-frequency distributions (Fig. 4B). When compared to the STFT spectrum of CT-PiezoADs, the spectra of the ST-PiezoADs exhibit different vibrational behaviors but show similar trends. Vibrational peaks are observed at low frequencies in channels 4 and 3, which have relatively larger radii and longer spring lengths. The resonant peaks shift to higher frequencies as the spring lengths decrease in channels 2 and 1.

The ST-PiezoAD features four separate channels that can also be connected in series to provide a single-channel output, where the upper or bottom electrodes are physically connected accordingly. To construct and analyze the tonotopic profile of the device, the piezoacoustic output of the single-channel configuration was recorded under frequency sweep scanning. The frequency characteristics of the ST-PiezoAD were then analyzed using STFT and peak analysis, as depicted in Fig. 4D. In the frequency analysis, voltage peaks were identified at frequencies of 655, 374, 240, 188, 142, and 93 Hz. These peak frequencies were further analyzed by Gaussian fitting functions. It is worth noting that the piezoacoustic peak of the single-channel output of the ST-PiezoAD aligns with the resonant peaks observed in each separate channel. This observation indicates that the ST-PiezoAD is capable of transducing and summarizing detailed sound information by capturing features across a wider range of frequencies through all four channels. This remarkable capability is attributed to the utilization of highly sensitive aligned piezoelectric nanofibers and the establishment of multiresonant coupling conditions.

To gain deeper insights into the coupled multiresonant behaviors of the ST-PiezoAD, the out-of-plane vibration was characterized using a laser vibrometer. The scanning point density was set to ~1.1 mm. The experimental mapping of the ST-PiezoAD (Fig. 4E) demonstrates clear separation of vibrational peaks along the radial directions of the fibrous membranes. In higher resonance modes, the vibrational modes observed in the experimental mapping became more intricate, as depicted in Fig. 4E. Specifically, the maximum displacement was observed at a low frequency of around 180 Hz in channel 4 with the largest radial (*d* = 60 mm). As the frequency increased to 200 Hz, the maximum displacement moved to channel 3. Those vibration peaks appear broad, spreading largely along the circumferential direction across the nanofiber membrane. As the frequency further increased to 320, 420, and 660 Hz, more vibrational peaks emerged and localized along the radial direction (highlighted by dashed loops), echoing the displacements measured at the center points of each channel (Fig. 4, A and B). Movie S1 showcases the dynamic vibrations of the ST-PiezoAD.

The device’s vibrational modes were further simulated using finite element analysis (FEA) modeling (Fig. 4F). On the basis of the observations in Fig. 2 (A and B), most of the nanofibers are aligned radially, while some cross over each other. Therefore, the fibrous membrane was considered as a continuous anisotropic elastic plate with radial Young’s modulus (*E*_1_ = 140 MPa) > circumferential Young’s modulus (*E*_2_ = 12 MPa), capable of transferring sound vibrations radially and transversely respectively across the channel. The simulated vibrational patterns demonstrate distinct vibration modes in response to the sound at the resonant frequency detected, resembling those detected to a large degree (Fig. 4E). Furthermore, the simulation results not only recapitulate the largest displacement in low vibration mode initially observed in channel 4 for mode 2 at low frequency (184.72 Hz) but also track the maximum vibration to propagate toward channels 3 and 2 in smaller diameters (233.3 Hz) as the frequency increased. The resonant vibrational peak at each channel followed the principle of the number of nodes increasing with increasing frequency while tended to split and spread into multiple elongated peaks along the radial direction with increasing frequency. This phenomenon can be observed in modes 4, 8, and 16 at frequencies of 294.85, 412.90, and 647.55 Hz, as depicted in Fig. 4F, similar to the radial localization patterns of vibration peaks in high frequency, as observed in the ST-PiezoAD.

It should be mentioned that simulated vibration peaks appeared broader, spreading in elongated oval patterns along the circumferential direction in low vibration mode at low frequency, which may be attributed to the uniformity of the continuous elastic plate with anisotropic elasticity (*E*_2_ < *E*_1_) defined in the FEA model compared to the less uniform aligned nanofibrous membrane with a porous structure. Because of the intrinsic nature of low stiffness of the polymeric nanofibrous thin film, primary broad vibration peaks in low frequency are bound to generate across the thin film with a stepped change of the radial. These findings align with our previously published work, where broad vibration peaks were observed in both isotropic and anisotropic nanofiber membranes under circular boundary conditions, depending on the fiber morphology, diameters of the device, and sound frequency (*34*). As the sound frequency increased, the radially localized peaks in high vibration modes in high frequency are mainly determined by the high radial elasticity (*E*_1_) of the aligned nanofibrous membrane. As such, the spiral shape and anisotropic piezonanofibers uniquely demonstrated the capacity of separating spectra of vibration and converting to the voltage output of the ST-PiezoAD in response to varying frequency, reminiscent of tonotopic function of the human auditory system.

### AI-assisted sound recognition

Trampoline-like piezoelectric acoustic devices have been demonstrated to exhibit outstanding multiresonant sound transduction, showcasing superior sensitivity and mimicking the tonotopic profile of the human auditory system. The piezoacoustic signal transduction carries rich information correlated to sound content and direction, which effectively emulate the functional information of both “peripheral and central auditory systems.” With the assistance of machine learning, an artificial “auditory cortex” model can be integrated into the device for a fully functioning hearing system.

We collected a comprehensive dataset comprising piezoacoustic signals from various directions and natural language content. For each direction, at least 200 piezoacoustic repeated scans were collected and used as inputs for training and testing procedures, as illustrated in fig. S4 (A and B). The device underwent more than 4800 test cycles over 2 weeks, demonstrating its robustness and repeatability during the tests. This dataset was used to train the piezoacoustic baseline model. The artificial “auditory cortex” is structured using machine learning algorithms, consisting of an “auditory spectrum encoder” and a “sound feature extractor” (fig. S4C), with the former designed to encode the input combined spectra as shallow features and the latter aiming to further extract the deep auditory features for the final prediction. After 150 iterations, the model achieved an accuracy of over ~90% on both training and validation data, indicating successful learning of patterns and effective generalization to unseen instances (figs. S4D and S4E).

**Fig. 5.**
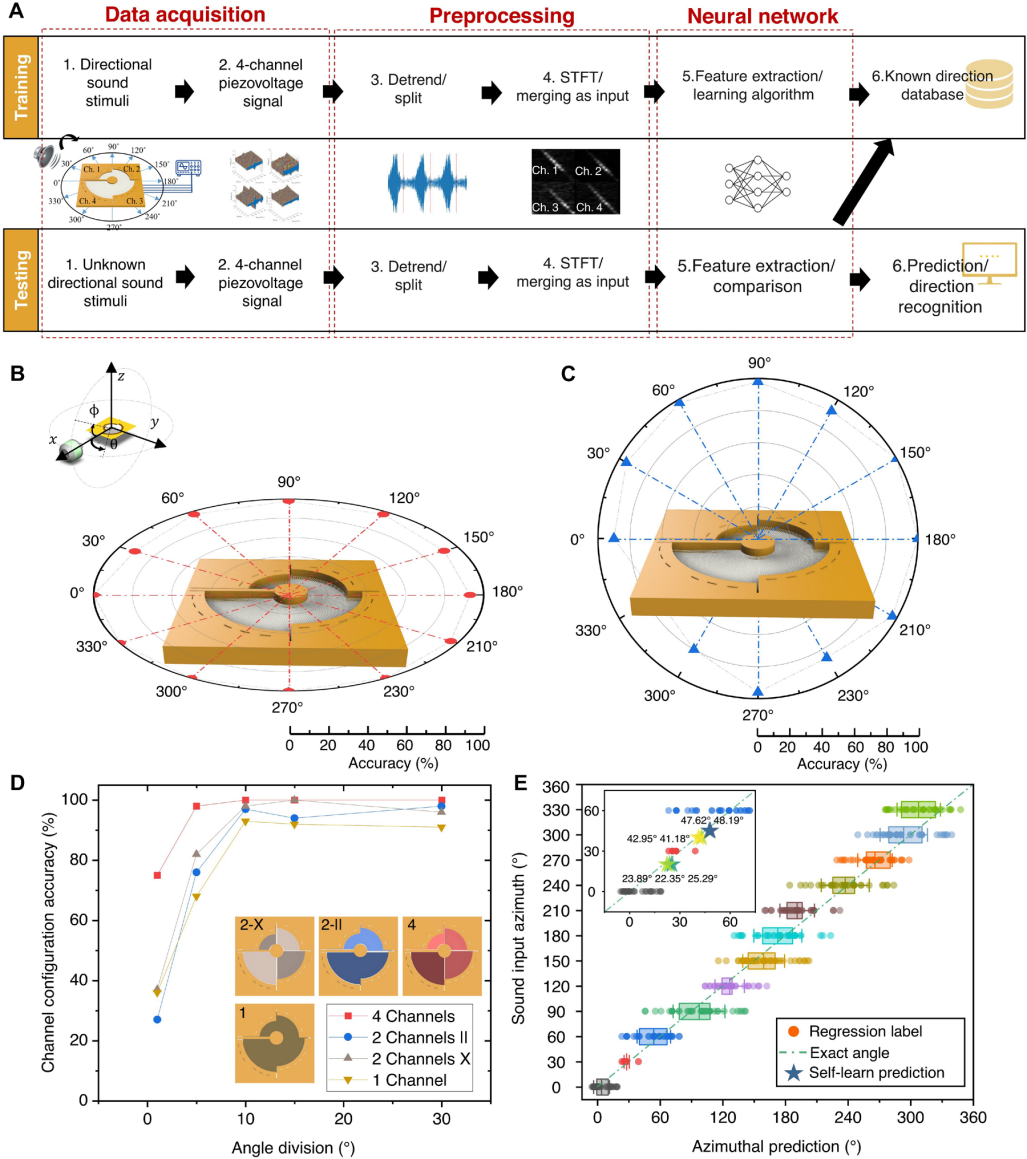
Neural network–assisted ST-PiezoAD for sound direction recognition. (**A**) Schematic workflow of the direction recognition model based on the ST-PiezoAD. The data acquisition phase involved the rotation of a mouth simulator around the device, generating sound sweeps (1500 to 100 Hz) at various angles (0° to 360°) in both horizontal and vertical planes. The transmitted piezoacoustic signals were preprocessed by detrending, splitting, and STFT; four-channel STFT spectra were merged as input for training and testing (image dimension, 256 by 256). (**B**) Recognition accuracy for incoming sound from θ on the *xy* plane and (**C**) recognition accuracy for incoming sound from ϕ on the *yz* plane (the speaker was set at 20 cm away from the center point with an SPL of 115 dB). (**D**) Classification-based sound recognition accuracy when increasing azimuthal step precision (minimum steps of 1°, 5°, 10°, 15°, and 30°) and based on different channels combinations. (**E**) Regression-based self-learn for unknown directions and prediction.

An attention-based transformer model was used to analyze essential directional features from ST-PiezoAD acoustic signals, namely the direction recognition model (Fig. 5A). During the “hearing training” procedure, all training data were fed into the proposed model by 200 iterations, with an additional 20 untrained data used for validation and 20 data points used for testing. The trained model was combined with the ST-PiezoAD as a whole artificial auditory module. The “hearing testing” procedure aims to evaluate the performance of the trained model, where the recognition was performed by comparing the unknown sound input features with the pretrained knowledge. The recognition accuracy is the ratio of correct predictions to the total testing number. The ST-PiezoAD module exhibited exceptional accuracy in direction recognition along the polar axis in 3D, covering a solid angle of 4π. Figure 5 (B and C) presents the classification-based recognition accuracy on the *xy* and *yz* planes, respectively, with an azimuthal step of 30°. The average accuracy for all 12 angles in the *xy* plane (θ) was 97 ± 3.2%, while in the *y* plane (Φ), it was 92.1 ± 8.1%. Moreover, the recognition accuracy for different distances along the *z* axis reached 100%, indicating the device’s sensitivity to changes in distance (fig. S5, A to D).

To investigate the effect of channel number on the recognition accuracy, the training data were collected from the ST-PiezoAD with one channel, two channels, and four channels, which physically combined the electrodes as illustrated in different colors in Fig. 5D. The azimuthal step precision of the artificial intelligence (AI)–assisted ST-PiezoAD module was investigated by training and testing with different stepwise angles of 1°, 5°, 10°, 15°, and 30°. The results show that the optimal accuracy of stepwise angles reduces when the moving step is smaller than 10°. Notably, the accuracy reduces considerably for the configurations with less channels. The recognition accuracy is always higher when using the ST-PiezoAD with a higher number of channels. In the two-channel configurations, it can be also noticed that the crossed channel configuration (X) shows higher recognition accuracy than that of paralleled channels (∥). This is related to the fact that the vibration of one channel affects the neighboring channels. The crossed channels reduced this effect by using the opposite channels, which was also one of the reasons to choose the attention-based model. A demo of the real-time recognition of sound directions was demonstrated with a pretrained ST-PiezoAD module. The rotational stage traveled to a random angle and played the sound to stimulate the ST-PiezoAD. A recognition was made and displayed on the screen (see movie S2). The overall accuracy of the real-time sound direction recognition is still outstanding at 96.0% (fig. S5, E and F).

The “auditory cortex” excels in classifying labeled sound directions, but it is not possible to supervise the training and exhaust all directions. Thus, we further developed a regression-based prediction to cover the complete range of the sound direction. The labels indicating the position of the sound source (0, 30, 60, …, 330) were hidden before feeding the data to the model. The box plot in Fig. 5E shows the performance of regression-based predictions, where 477 unlabeled data from the *xy* plane were used to test the model and request for a prediction of any angles between 0° and 360°. Notably, the labels of these sound directions are distributed close to their exact direction angles. Notice that the *R*^2^ value (*52*) of the results is 0.93, which also prove the good training quality of regression-based prediction. To prove the self-learning ability of the model, it is expected to recognize the angles that have never been labeled or trained, such as 22°, 40°, and 45°. The piezoacoustic signals were collected from these unknown, untrained, and unlabeled directions and required the prediction of their detailed directions. The regression-based model responded with reasonable predictions that were close to the exact angles (Fig. 5E, inset).

To demonstrate the capability of the “auditory module” based on PiezoADs for future voice interface applications, we further extended the applications to sound understanding tasks such as sound recognition (figs. S6 and S7 and movie S3), as well as music recording. The device exhibited accurate sound recognition by analyzing famous “Hamlet” excerpts. In addition, high-quality music recordings were demonstrated, wherein the device emitted sound through a speaker, recorded the music using piezoelectric signals, and reconstructed it for playback (movie S4).

## DISCUSSION

An artificial hearing system has been developed by combining a programmable asymmetric structure of piezoelectric nanofibers with digitally intelligent neural networks, resembling the processing stages of the human auditory system. A spiral trampoline–like acoustic device platform based on well-aligned piezoelectric nanofibers demonstrates exceptional functions in sound recognition and directional recognition with excellent sensitivity and accuracy. The asymmetric spiral geometry of the piezoelectric nanofibrous membrane not only exhibits complex multiresonant vibration spectra, effectively mimicking the vibrational transduction and frequency separation of the basilar membrane in the human cochlea, but also mimics and even outperforms the spatial intelligence of the human brain in assisting with neural networks. The AI-assisted ST-PiezoAD demonstrates outstanding accuracy in recognizing sound in all directions. Moreover, increasing the number of channels in the ST-PiezoAD improved the accuracy of sound and direction recognition, highlighting the potential for further devices to expand the channel count based on nanofiber springs and nanoscale electrodes close to the continuous spiral shape of the basilar membrane. Miniaturization of the devices, system integration, and field tests are the next indispensable steps for paving the way toward real-world applications. The development of a piezoelectric nanofiber–based intelligent hearing system represents a paradigm shift in the fields of artificial cochlear devices, artificial auditory systems, and artificial spatial intelligence. The system offers multiple auditory functions, bringing us closer to replicating the capabilities of the human hearing system.

## MATERIALS AND METHODS

### Design and fabrication of the trampoline-like PiezoADs

The methodology for the fabrication of the flexible PiezoADs involved the use of AutoCAD for design and the utilization of polyimide (PI) films and copper sheets. The trampoline-like shape with concentric patterns was laser cut from a flexible PI sheet with a thickness of 0.5 mm. Subsequently, heat pressing was performed to attach the copper electrode onto the PI film. It is important to note that all the electrodes in each device with different diameters were designed to have the same electrode area. To ensure stability and facilitate the performance tests, an acrylic sheet was used as a holder. This holder effectively secured the sandwiched structure of the device in place, enabling it to maintain stability during the resonance vibration tests.

### Fabrication of radially aligned PVDF-TrFE/BTO NP nanofibers

The P(VDF-TrFE) powder (Piezotech FC25, 75/25 mol %, France) was dissolved in a mixture of acetone (Sigma-Aldrich) and dimethylformamide (DMF) in a 2:2 volume ratio. The solution was stirred overnight at 100 rpm to ensure thorough mixing. BaTiO_3_ nanoparticles (Sigma-Aldrich, 467634, particle size <100 nm, UK) were suspended in absolute ethanol and modified with 0.5 wt % 3-aminopropyltriethoxysilane (Sigma-Aldrich, 440140, 99%). The suspension was vigorously stirred for 12 hours at 25°C and then washed extensively with distilled water. The surface-treated BTO NPs were redispersed in a DMF solution and stirred for 12 hours, followed by 1 hour of sonication to achieve a homogeneous dispersion.

The presolutions of PVDF-TrFE and BTO NPs were then mixed and stirred overnight. Subsequently, they were sonicated for at least 2 hours before use. The resulting solution contained 20 wt % PVDF-TrFE in an acetone/DMF ratio of 2:3, and the weights of the BaTiO_3_ NPs were 0, 2, 4, and 6 wt % of the weight of PVDF-TrFE. After the final stirring, the solution was loaded into a 10-ml syringe for electrospinning. The syringe was connected to a 14-gauge steel needle using a polytetrafluoroethylene peristaltic pump tubing (Adhesive Dispensing Ltd.). The needle was inserted into a custom-built electrospinning machine connected to a dc voltage power supply (Glassman High Voltage Inc.), with the voltage set at 15 kV. The syringe was placed on a syringe pump (World Precision Instruments, US) with a constant output pumping rate of 1 ml/hour.

The cantilever-type device was positioned at 15 cm below the needle, and all electrodes were grounded. The device was rotated at a speed of 30 rpm during the electrospinning process. The needle holder moved parallel to the device at a speed of 5 cm/s to ensure uniform electrospinning. All electrospinning procedures were conducted at room temperature (22° to 25°C) and ~50% humidity, with the aid of a central ventilation system and a dehumidifier.

### Characterization of the nanofibers

The morphology, structure, and ferroelectric properties of PVDF-TrFE/BTO nanofibers were analyzed using various characterization techniques. Scanning electron microscopy (ZEISS EVO MA10), AFM, X-ray diffraction, and PFM were used for this purpose. X-ray diffraction patterns were obtained using a 2D Vantec (Brooker, Germany) instrument with a Cu Kα x-ray source (λ = 1.54 Å), operating at 50 kV and 1 mA. Data collection was performed in the 2θ range of 5° to 70° with a step size of 0.05°.

AFM was operated in tapping mode using an MFP-3D system (Asylum Research, Santa Barbara, CA) using conductive cantilevers with an approximate spring constant of 2.9 N/m and a quality factor of 16 to 40. The cantilevers were driven by a voltage amplitude of ~1.2 V, and the scan frequency was set at 0.3 Hz. Fast force mapping mode was used to assess the nanomechanical properties of the nanofibers. The experimental data for the tip-sample contact were fitted using the Derjaguin-Muller-Toporov model. The ferroelectric properties of a single fiber were characterized using PFM with the dual ac resonance tracking PFM technique. The PFM cantilever was tuned to a resonance frequency of 270 kHz, and a maximum bias voltage of ±60 V was applied.

To investigate the mechanical properties of the PVDF-TrFE/BTO nanofibers, a tensile test was conducted. Nanofibers were collected and rolled into a cylinder, with most fibers aligned along the longitudinal direction. The dimensions of the cylinder were measured using bright field microscopy. The tensile test was performed using an Instron 5565 (Instron, UK) equipped with a 2525 Series Drop-Through Static Load Cell (50 N). The cylinders were clamped and stretched along the direction of the fibers at a rate of 5 mm/min until complete failure occurred, while force and strain were recorded. Five repetitions of the test were performed for nanofibers with BTO concentrations of 0, 2, 4, and 6 wt %.

### Piezoperformance test

A custom piezoacoustic laser-vibrometer system was assembled using a laser vibrometer (MSA-050 Microsystem Analyzer, Polytech, Germany), an acoustic speaker (Type 4227-A, Brüel & Kjær, Denmark), and high-impedance JFET input voltage buffers connected to a data acquisition device (Powerlab 16, ADInstruments). The laser vibrometer was used to measure the vibration of the PiezoAD device in response to an acoustic stimulus. The Powerlab LabChart program was used to capture the displacement and voltage signals at a sampling frequency of 40 kHz. The acoustic speaker was positioned at various distances below the devices, and the SPL was set to 94, 108, and 114 dB, corresponding to ~1, 5, and 10 Pa, respectively.

### FEA of the vibrational mode of ST-PiezoAD

To mimic the anisotropic nature of the aligned nanofibers, a finite mesh of ST-PiezoAD was defined with varying radial and circumferential Young’s moduli. Hybrid meshing techniques were used to ensure precise control of the meshes, aligning with the local radial geometry. On the basis of the mechanical properties of the different materials, the nanofiber area was defined with a radial Young’s modulus of *E*_1_ = 140 MPa and a circumferential modulus *E*_2_ = 12 MPa and the boundary PI films were defined with *E*_3_ = 10 GPa. The intrinsic vibrational modes were then analyzed accordingly.

### Data processing and machine learning

In this work, three AI-based models have been designed to assist the nanofibers: piezoacoustic baseline model, direction recognition model, and speech recognition model. Python 3.7 was used to process sound direction recognition experiments, including preprocessing of piezoacoustic signals, visualization of spectrogram images, and the ML-model construction and training (operated under Windows 11).

We collected a comprehensive dataset comprising piezoacoustic signals from various directions and natural language content. This dataset was used to train the piezoacoustic baseline model. The input piezoacoustic signal was first “detrend” by standard scripy from Python library “scip.signal.detrend,” which removed the linear bias/trend. The splitting of data was conducted by seeking the periodic peaks with a similar feature. STFT was then performed on the data to convert the time-dependent voltage signals to spectrogram images. Hanning window was used with a window length of 4096 and a hop size of 256.

For the direction recognition model, we used an attention-based transformer machine learning model. The model, which mimics the structure of the auditory cortex, consists of two main components: an “auditory spectrum encoder” and a “sound feature extractor.” These components were implemented using machine learning algorithms to effectively process and analyze the input data. The auditory spectrum encoder is made by convolutional neural networks (*53*), where we set residual connections (*54*) in each block to alleviate auditory information loss during feature encoding. The sound feature extractor exploits the attention mechanism (*55*) to deal with deep auditory features, including a formal attention layer and three self-attention–based vision transformer layers, helping the model focus more on the important STFT-channeled information by setting different feature weights. The outputs are different according to the selected strategy, where classification-based prediction outputs the categories (0, 30, 60, …) and regression-based prediction can output any possible value between 0° and 360°.

For applying classification-based prediction, the final layer of the ML model is a linear layer with *n* nodes, which can provide the confidence of each category, where *n* refers to the number of data categories. Then, the category with the largest confidence is the final prediction result. The training loss of this strategy is cross-entropy loss (*56*). The loss of classification-based prediction was defined as

$$L_{\text{cls}}=-\frac{1}{n}\sum_{i=1}^{n}y_{i}*\text{log}\left({\hat{y}}_{i}\right)$$

where $L_{\text{cls}}$ refers to the loss of classification-based prediction, *n* is the number of data categories, $y_{i}$ is the true label of data, and ${\hat{y}}_{i}$ is the predicted label.

For applying regression-based prediction, the final layer of the ML model is a linear layer that can directly provide the predictive value. The training loss of this strategy is mean squared error loss (*57*). The loss of regression-based prediction was defined as

$$L_{\text{reg}}=\frac{1}{k}\sum_{i=1}^{k}{\left({\hat{y}}_{i}-y_{i}\right)}^{2}$$

where $L_{\text{reg}}$ refers to the loss of regression-based prediction, *k* is the number of training samples, $y_{i}$ is the true value of data, and ${\hat{y}}_{i}$ is the predicted value.

The capability of the AI model was extended to include sound content recognition. This involves analyzing the acoustic signal to identify and differentiate between various types of sound content.

### Statistical analysis

Data presentation and statistical analyses were performed in Origin (version 2020b, OriginLab Corporation, Northampton, MA) and MATLAB (version 2021b, The MathWorks Inc., Natick, MA). Custom scripts written in MATLAB were developed to process and split the data acquired from the experiments. All descriptive data are presented as the means ± SD.

## Supplementary Materials

### The PDF file includes:

Supplementary Text Figs. S1 to S7 Tables S1 and S2 Legends for movies S1 to S4 References
