# Supplementary material for: Piezoelectric nanofiber–based intelligent hearing system
Source: Sci Adv. 2025 May 7;11(19):eadl2741. doi: 10.1126/sciadv.adl2741 (PMC12057686; doi:10.1126/sciadv.adl2741)
Supplement: Supplementary file 1 — Supplementary Text Figs. S1 to S7 Tables S1 and S2 Legends for movies S1 to S4 References [file sciadv.adl2741_sm.pdf]

Science Advances
AAAS

Supplementary Materials for

Piezoelectric nanofiber–based intelligent hearing system

Jinke Chang *et al.*

Corresponding author: Wenhui Song, w.song@ucl.ac.uk

Sci. Adv. **11**, eadl2741 (2025)
DOI: 10.1126/sciadv.adl2741

The PDF file includes:

Supplementary Text
Figs. S1 to S7
Tables S1 and S2
Legends for movies S1 to S4
References

Other Supplementary Material for this manuscript includes the following:

Movies S1 to S4

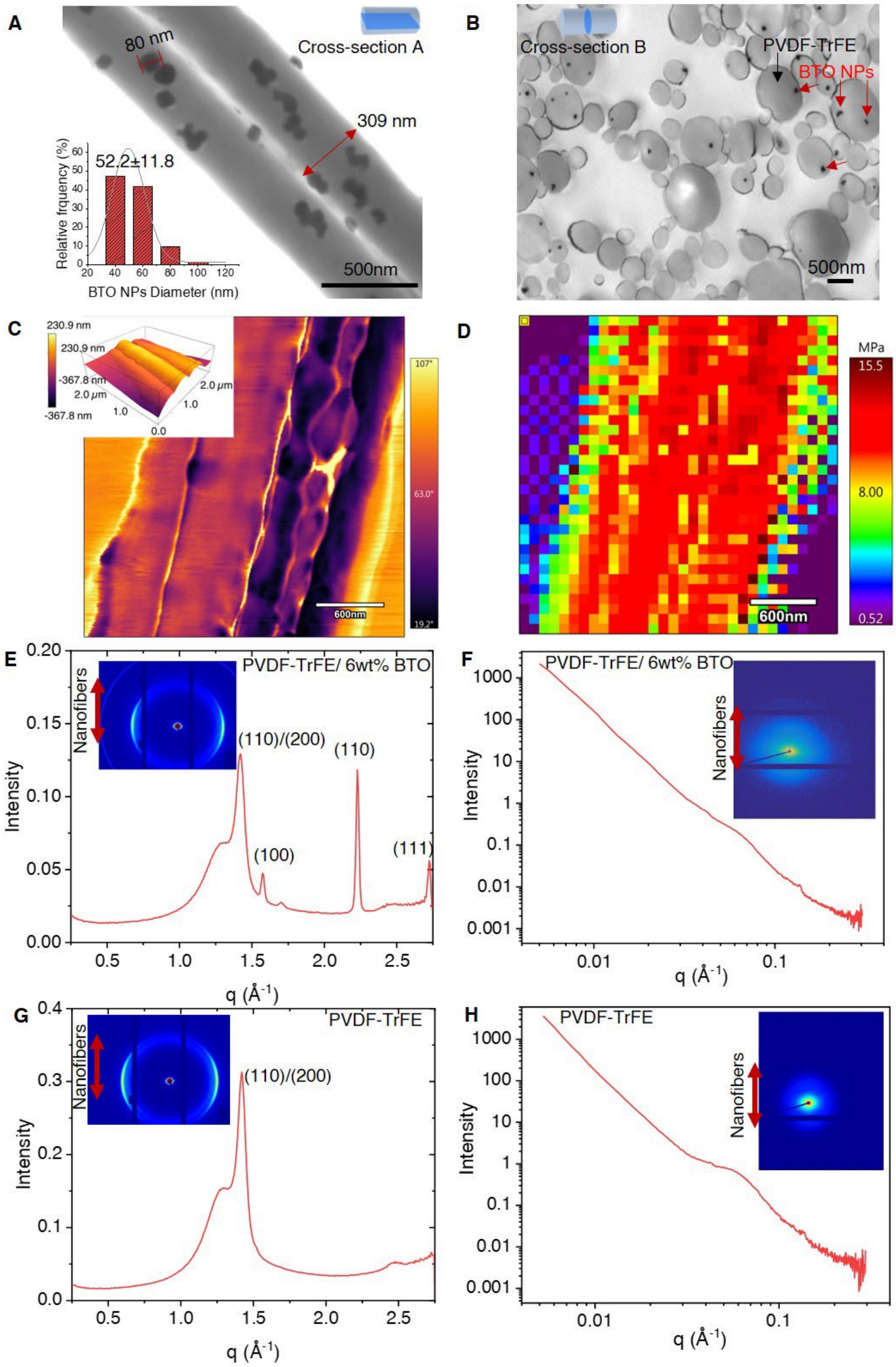

A
80 nm
Cross-section A
309 nm
52.2±11.8
Relative frquency (%)
BTO NPs Diameter (nm)
500nm
B
Cross-section B
PVDF-TrFE
BTO NPs
500nm
C
230.9 nm
-367.8 nm
2.0 μm
1.0
0.0
600nm
107°
63.0°
19.2°
D
MPa
15.5
8.00
0.52
600nm
E
PVDF-TrFE/ 6wt% BTO
Nanofibers
(110)/(200)
(110)
(100)
(111)
Intensity
q (Å-1)
F
PVDF-TrFE/ 6wt% BTO
Nanofibers
Intensity
q (Å-1)
G
PVDF-TrFE
Nanofibers
(110)/(200)
Intensity
q (Å-1)
H
PVDF-TrFE
Nanofibers
Intensity
q (Å-1)

Fig. S1. Characterisation of PVDF-TrFE/BTO nanofibers. (**A**), STEM of the longitudinal cross-section and of PVDF-TrFE/6wt% BTO NPs nanofibers (inset: BTO nanoparticle diameter measured by dynamic light scattering). (**B**), STEM of the transverse cross-section of PVDF-TrFE/6wt% BTO NPs nanofibers. (**C**), 3D height, phase measurement and (**D**), Force mapping of PVDF-TrFE/BTO nanofibers using atomic force microscopy. (**E**), 1D WAXS profile of P(VDF-TrFE)/6wt% BTO nanofiber obtained from the integration of 2D WAXS pattern in the inset. (**F**), 1D SAXS profile of the P(VDF-TrFE)/6wt% BTO nanofiber obtained from the integration of the 2D SAXS pattern in the inset. (**G**), 1D WAXS profile of P(VDF-TrFE) nanofiber obtained from the integration of 2D WAXS pattern in the inset. (**H**), 1D SAXS profile of the P(VDF-TrFE) nanofiber obtained from the integration of the 2D SAXS pattern in the inset.

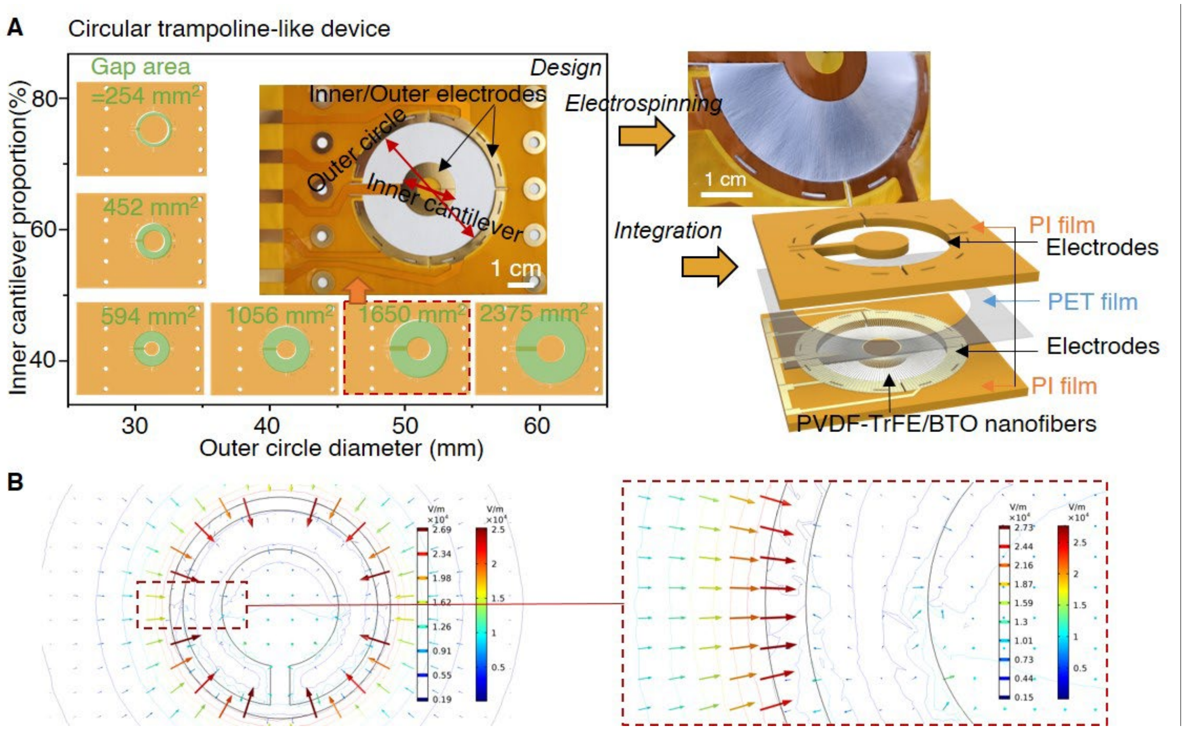

Fig. S2. Design and fabrication of the Piezoacoustic devices (**A**), Design, electrospinning and integration of the circular trampoline-like device with different inner cantilever proportion and outer circle diameter (Inner cantilever proportion is defined as the ratio of inner diameter and outer diameter). (**B**), electric filed simulation of the circular trampoline-like device under electrospinning conditions at applied voltage of 15 kV, needle-to-electrode distance 15 cm.

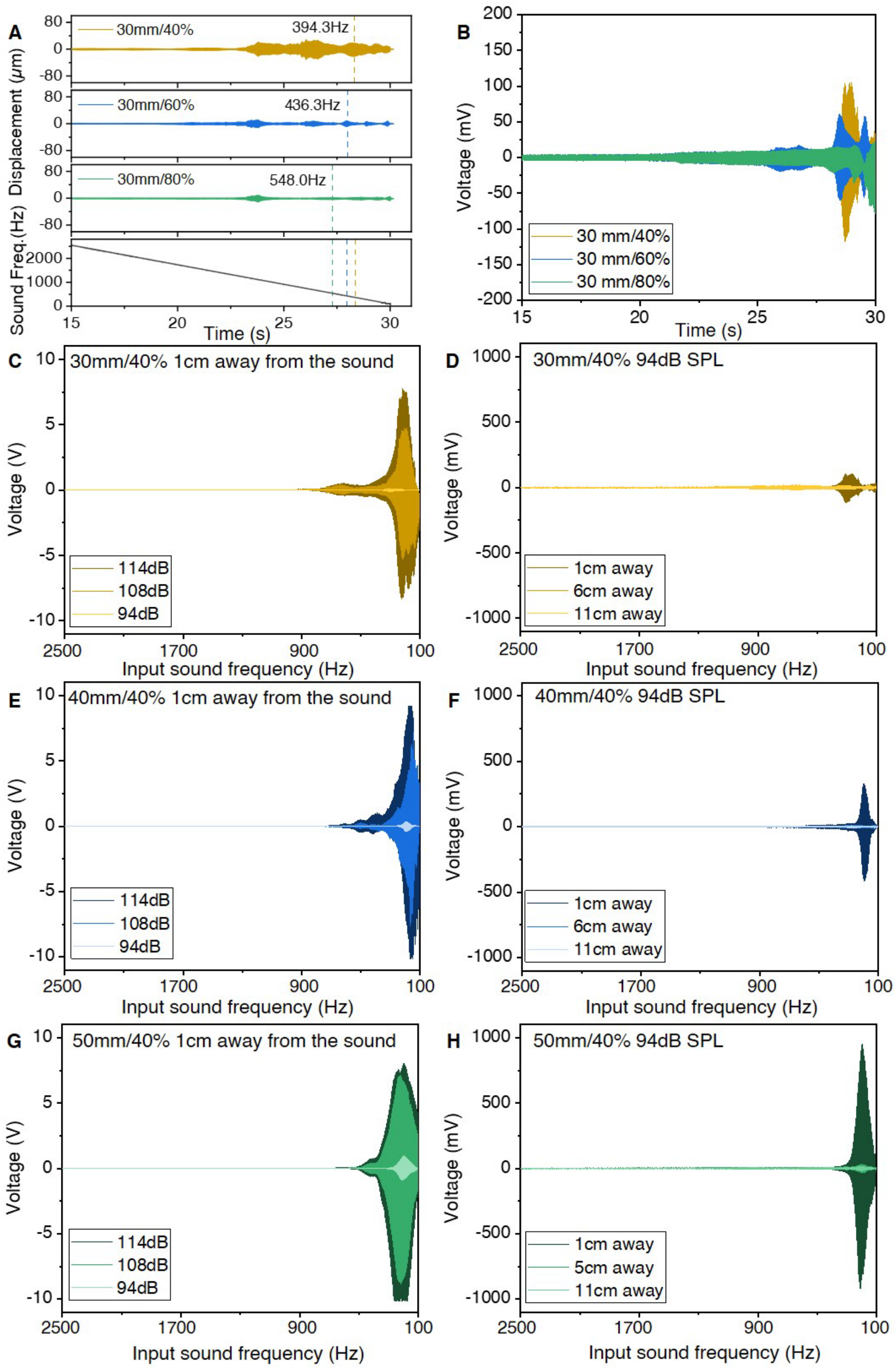

Fig. S3. Piezoacoustic transduce performance of CT-PiezoADs. (**A**), Displacement of 30 mm devices with cantilever proportion of 40%, 60% and 80%; (**B**), voltage output of 30 mm devices with cantilever proportion of 40%, 60%, 80%, with SPL of 94 dB and 1cm away from the speaker; Voltage responses of CT-PiezoADs with geometry of (**C, D**), 30mm/40% (**E, F**), 40mm/40% and (**G, H**), 50mm/40% under SPL of 94 dB, 108 dB and 114 dB and 1 cm away from the speaker, or under standard SPL of 94 dB and 1 cm, 6 cm, 11 cm away from the speaker.

Table S1. Designs and Performances of Piezo/triboelectric acoustic sensors in the literature.

Materials	Size	Frequency range	Sensitivity (mV/Pa)	Functions	ML model	Principle	Ref.
P(VDF-TrFE)) /BaTiO3 particles	80 mm *80 mm * 0.46 mm fabric	80-1000 Hz	19.6	Acoustic sensing	n/a	Piezoelectric	*(35)*
P(VDF-TrFE)	1.6 mm *0.4 mm * 2000 mm	2-8 MHz	100 mV	Fibre sensor array3*3 and acoustic positioning	n/a	Piezoelectric	(*58*)
Polytetrafluoroethylene (PTFE)/ graphite	40 mm *40 mm, thickness of ≈0.35 mm	0.1–20 kHz	3.2	Speech recognition, voice signature, voice controlling	n/a	Triboelectric	(*59*)
ZnO nanowire		<250Hz	n/a	Acoustic sensing	n/a	Piezoelectric	(*60*)
PVDF	1200 mm^2	0.18-2 kHz	266	Acoustic sensing	n/a	Piezoelectric	(*50*)
Fluorinated ethylene propylene	15 mm* 75 mm *75 mm; gap, 0.2 mm	0.1-5 kHz	110	Music recording, Voice recognition, hearing aid	n/a	Triboelectric	(*47*)
Ag nanowires	A few centimetres,	0.1-10K	n/a	Voice recognition for personal voice security system	n/a	Triboelectric	(*61*)
SU-8	<9 mm^2	0.015–10 kHz	100	Voice recognition	n/a	Sophisticated capacitive	(*62*)
Lead-zirconate-titanate (PZT)	130 mm^2	0.1- 4 kHz	52	Voice recognition	Gaussian mixture model (GMM) algorithm	piezoelectric	(*36*)
Nb-doped PZT	0.173 mm^3	0.1 -8 kHz	30	Authenticated speaker recognition	multi-channel attention CNN	Piezoelectric	(*63*)
PVDF film	799 mm^2	1.4–4.9 kHz	0.0253	n/a	n/a	Piezoelectric	(*40*)
Aluminium nitride film	7.05 mm^2	2.6–13.3 kHz	1.67	n/a	n/a	Piezoelectric	(*64*)
PVDF-TrFE nanofibers	1.8 cm^2	0.200–5 kHz	71	Acoustic sensing	n/a	Piezoelectric	(*65*)
PVDF nanofibers	25 mm*25 mm	<500 Hz	10,050.6	Heart vibration	n/a	Triboelectric	(*66*)
PZT	100 mm^2	<240 Hz	~250	Acoustic sensing	n/a	Piezoelectric	(*67*)
Polyacrylonitrile nanofiber membrane	30 mm*40 m m*30 µm		23,401	Sound recognition	n/a	Triboelectric	(*68*)
PVDF/ZnO nanofiber membrane	100 mm *35 mm	<200 Hz	3,000	Acoustic sensing	n/a	Triboelectric	(*69*)
PVDF-TrFE/BTO NPs nanofibers	7.5 cm2 effective electrode area	<1500 Hz	1532.9	Direction prediction, sound recognition	Attention-based model	Piezoelectric	This work

Table S2 Piezoelectric coefficients of piezoelectric materials

Piezoelectric Materials	Structure	Processing techniques	Piezoelectric coefficient, d33/d31	Measurement methods	Ref.
PVDF-TrFE/BTO	Film	3D printing/ electric poling	-16±0.55 pC/N (d_{33}, PVDF-TrFE)<br>-20 ±0.6 pC/N (d_{33}, composites)	Conventional quasistatic method	(*70*)
PVDF-TrFE/BTO	Belt-like Fibres	Thermal drawing	46 pC/N (d_{31}, composites)<br>20 pC/N (d_{31}, PVDF-TrFE)	Conventional quasistatic method	*(35)*
PVDF-TrFE/BTO	Film	Spin coating and poling	-23 pC/N (d_{33}, composites)<br>-21 pC/N (d_{33}, PVDF-TrFE)	Conventional quasistatic method	(*71*)
PVDF-TrFE/BTO	Fibrous film	Electrospinning/ electrospray	32.72 pC/N (d_{33}, composites)<br>12.40 pC/N (d_{33}, PVDF-TrFE)	Conventional quasistatic method	(*72*)
PVDF-TrFE/BTO	Film	Solvent casting/ electric poling	20 pC/N (d_{33}, composites)<br>5 pC/N (d_{33}, PVDF-TrFE)	Conventional quasistatic method	(*73*)
PVDF-TrFE/BTO	Film	Spin coating	146±7.31 pC/N (d_{33}, composites)<br>21±1.36 pC/N (d_{33}, PVDF-TrFE)	Piezoelectric force microscopy (PFM)	(*74*)*
PVDF-TrFE/BTO	Nanofiber membrane	Electrospinning	21.2 pC/N (d_{33}, composites)<br>191 pC/N (d_{33}, BTO)<br>7.3pC/N (d_{33}, PVDF-TrFE)	Piezoelectric force microscopy (PFM)	(*75*)*
PVDF-TrFE	Film	Imprinting and electric poling	18–24 pm/V (d_{33})	Piezoelectric force microscopy (PFM)	(*76*)*
PVDF-TrFE	Film	Spin coating	1.195 pm/V (d_{33})	Piezoelectric force microscopy (PFM)	(*77*)*
BTO	Nanoparticles	3D printing and electric poling	160 pC/N (d_{33})	Conventional quasistatic method	(*78*)
BTO	Nanowires	hydrothermal reaction	43 ± 2 pm/V (d_{33})	Piezoelectric force microscopy (PFM)	(*79*)*
PVDF-TrFE/BTO	Nanofibre	Electrospinning	173 pC/N (d_{33}, composites)<br>20.9 pC/N (d_{33}, PVDF-TrFE)<br>181.0 pm/V (BTO)	Piezoelectric force microscopy (PFM)	This work*

Note: *It should be mentioned that the measured coefficient using PFM only represents the local piezoelectric responses in nanoscale area under a specific condition. Therefore, it may not be comparable with d_{33} values of bulk samples measured by conventional methods. We should also note that in studies by experts in this field, PFM poses significant challenges for the quantitative measurement of piezoelectric constants (*T. Jia, et al. Appl. Phys. Rev., 5, 021102, 2018*).

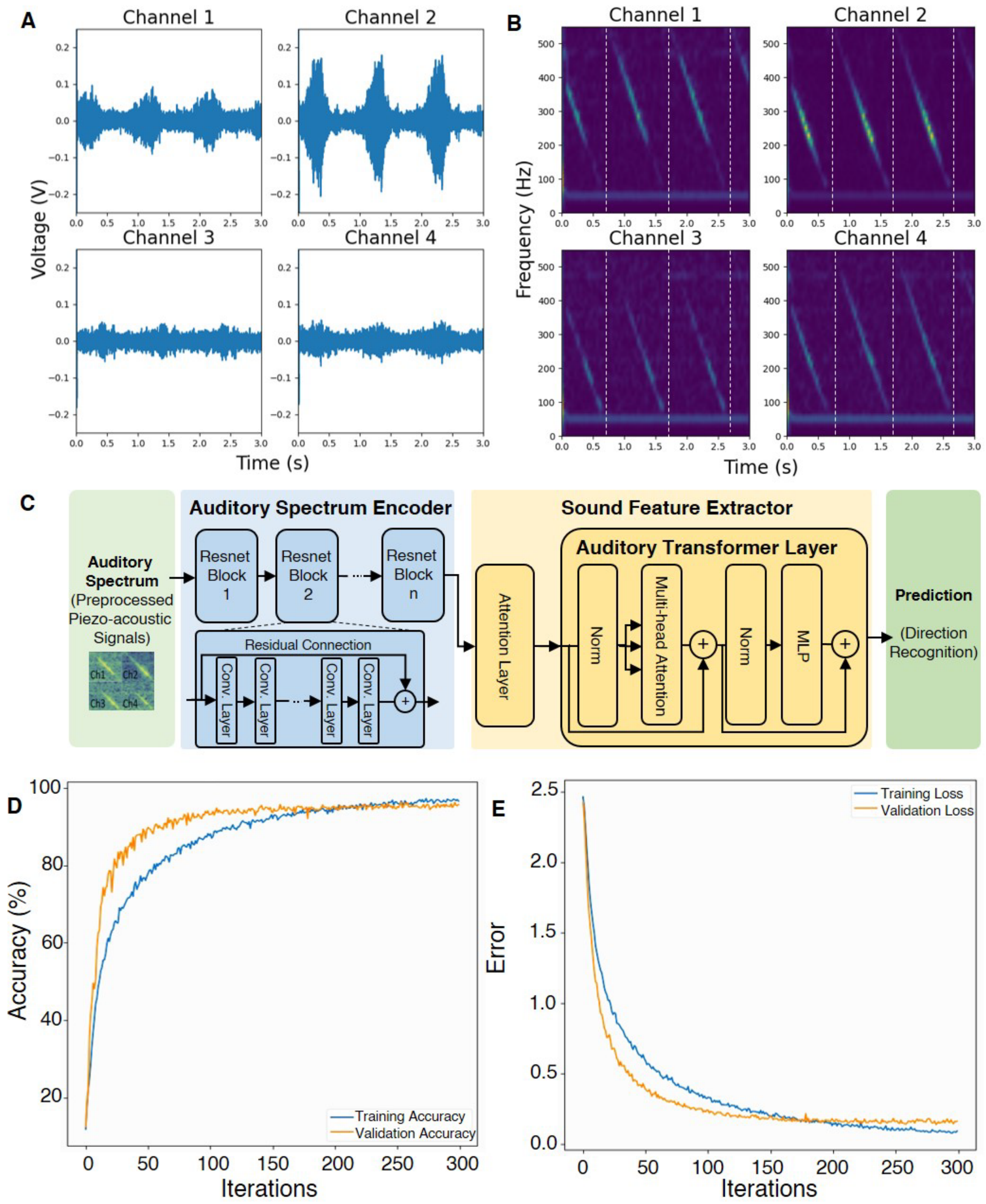

Fig. S4. Data acquisition, pre-processing and machine learning model of ST-PiezoAD. (**A**), an example of 4-channel voltage signals of ST-PiezoAD in response to sound stimulation from 0°. (**B**), spectrum of 4-channel signal outputs using STFT. (**C**), structure of machine learning prediction and regression based on auditory spectrum encoder and sound feature extractor. (**D**), Training accuracy and validation accuracy of the neural network. (**E**), training loss and validation loss during the training process

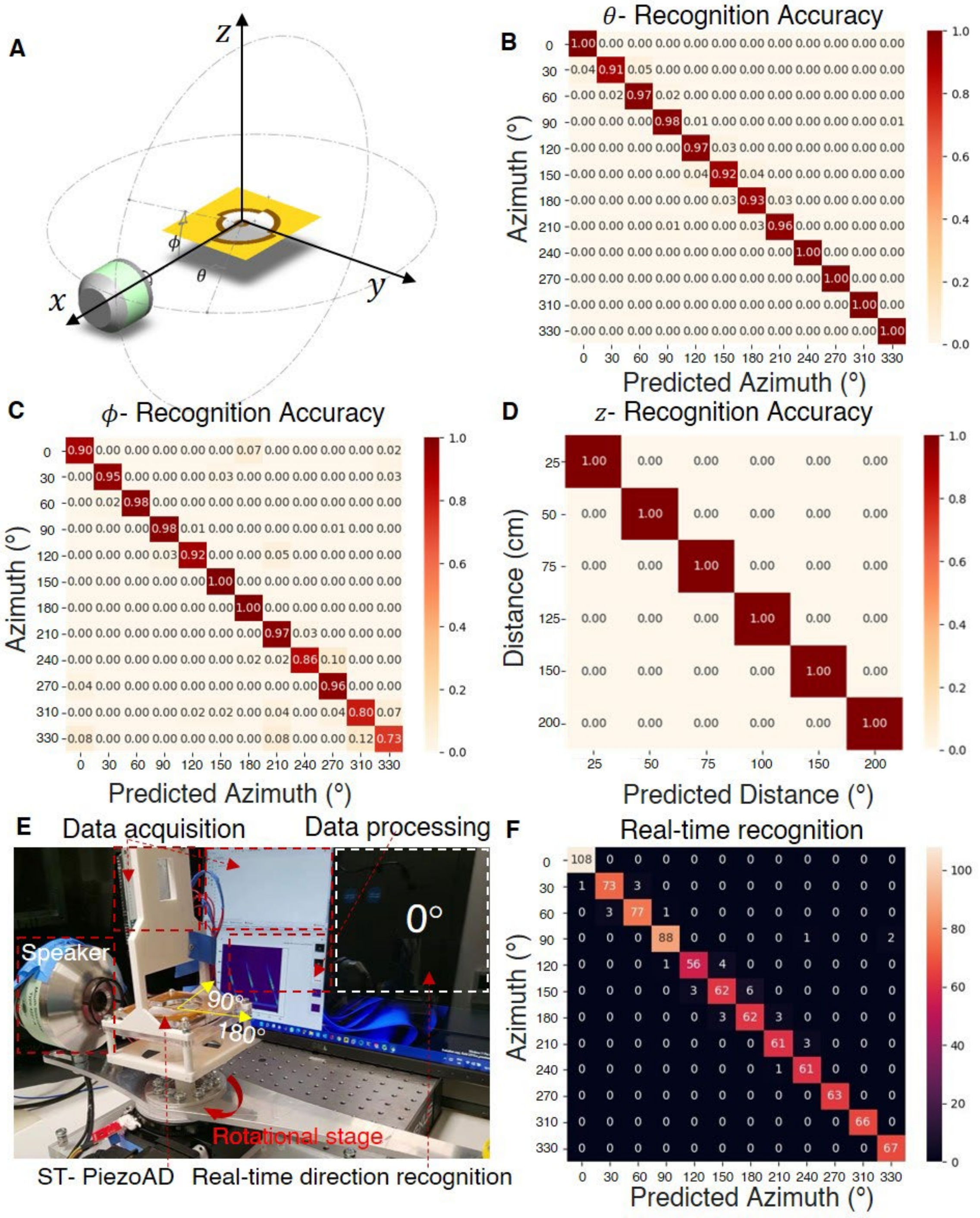

Fig. S5. Direction recognition accuracy based on St-PiezoAD. (**A**), Defined sound input directions in polarised axis. (**B**), direction recognition accuracy in XY plane, θ. (**C**), direction recognition accuracy in XZ plane, φ. (**D**), direction recognition accuracy of changing distances in Z direction. (**E**), photograph of a real-time direction recognition prototype. (**F**), confusion matrix of accuracy for the real-time sound direction recognition.

A CT-PiezoAD was used to demonstrate the speech recognition capability. A speech voice database (Hamlet – To be or not to be, Act 3 Scene 1) was used to stimulate CT-PiezoADs and generate a series of voltage signals and stored in a database accordingly. The audio data was downloaded from Librivox public domain licenses. Six sentences are used in the recognition as a proof of concept. The voltage signals (responding to the full sentences) were then used in pre-processing of the training, where the replicated signals were detrended and split. The voltage signals as a function of time were converted to the frequency domain using a short-time Fourier time (STFT) function and merged as 2D inputs for classification-based training. As the model was trained and validated until full maturity, a known speech voice database was formed. During the test workflow, random speech voice was given to the CT-PiezoAD (Fig. S6). Same data acquisition, pre-processing and the feature extraction process was performed, and the features were used to compare with the known database. A prediction was made and exported to a monitor displaying real-time speech content. An example of the data acquisition and pre-processing based on a 30mm/40% CT-PiezoAD is shown in Fig. S6A. The original voice signal "To be or not to be" was fed to the CT-PiezoAD to be converted into voltage signal as a function of time, as shown in Fig. S6B. For pre-processing, the data was automatically split into single sentences by analyzing the peak distributions (Fig. S6C). Separated voltage signals were converted to the frequency domain and merged with the original waveform as 2D black-and-white inputs of the training, as shown in Fig. S6D and S6E. The training accuracy and loss were displayed in Fig. S7, A and B. Accuracy reached 99.99% with the loss decreasing to 0.01 after only 20 iterations, which showed the consistency and repeatability of the piezoacoustic data acquired from the CT-PiezoADs. Fig. S7C illustrates the speech contents used for the training. Similar training processes were performed for all the speech voices until a known speech voice database was formed and the CNN reached a proper maturity. The set-up of the training and testing process is displayed in the photograph (Fig. S7D). During the testing process, random speech voices that were selected from the training database were given to the CT-PiezoAD module. It recognized the speech voices by comparing the features with the existing database. A prediction was made and displayed on the monitor. As shown in Fig. S7E and S7F, the average accuracy of the speech content recognition is up to 99.17%. A real-time speech recognition was demonstrated in the Supplementary Video 3, showing an outstanding accuracy of 91.33%. Our system shows promising accuracy in a well-controlled lab environment which is proof of the concept of using the PiezoAD for speech recognition.

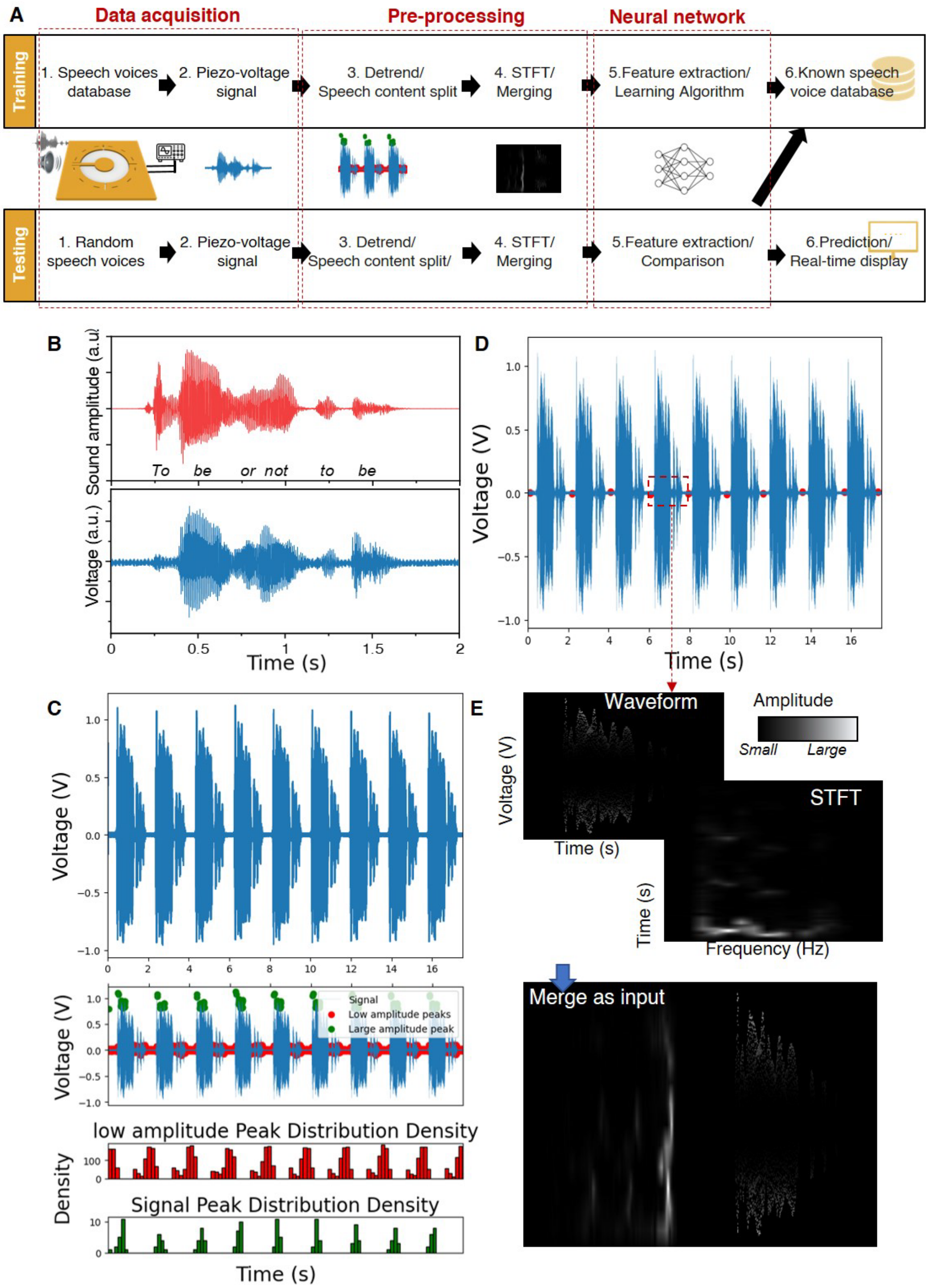

Fig. S6. Speech signal acquisition and pre-processing based on 30mm/40% CT- PiezoAD. (**A**), Schematic workflow of speech recognition. (**B**), Original sound amplitude- "To be or not to be", and corresponding piezoelectric voltage signal based on CT- PiezoAD device. (**C**), Input voltage signal repeats and peak distribution. (**D**), automatic speech sentence segmentation. (**E**), data pre-processing based on merged waveform and STFT plots, used as inputs of CNN deep learning algorithm.

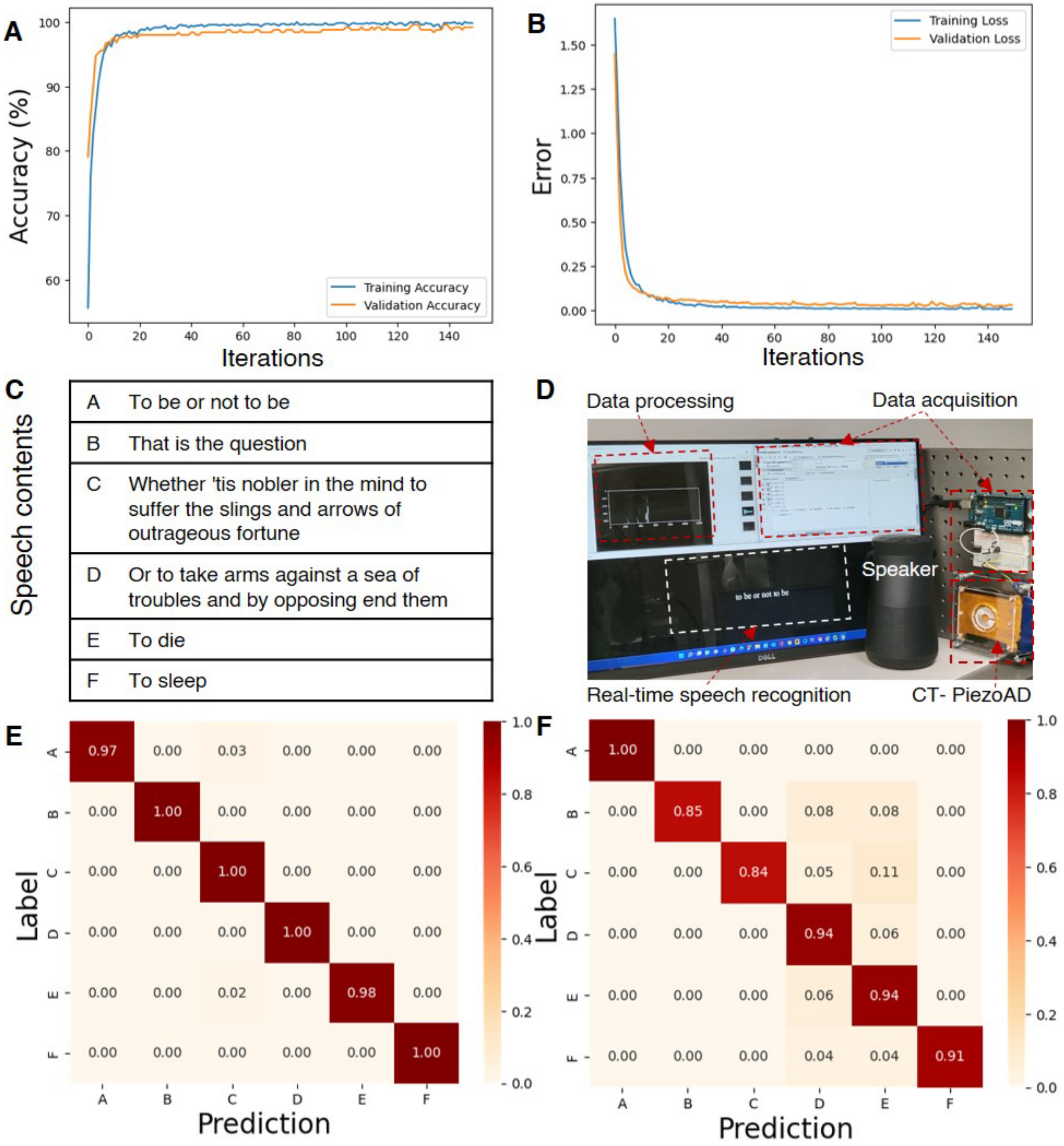

Fig. S7. Speech recognition training and real-time recognition test. (**A**), Training and validation accuracy. (**B**), Training and validation loss. (**C**), speech contents used for training of the neural network and real-time recognition test. (**D**), Photograph of real-time speech contents recognition test based on CT- PiezoAD device. (**E**), prediction matrix based on training data for neural network adjustment, inputs number N =600. (**F**), Prediction matrix for blind tests of real-time recognizing speech contents in "Hamlet", inputs number N=600.

REFERENCES AND NOTES

1. C. J. Plack, *The Sense of Hearing* (Routledge, 2018).

2. S. M. Echteler, R. R. Fay, A. N. Popper, Structure of the mammalian cochlea. *Comp. Hear. Mammals* **4**, 134–171. (1994).

3. A. Hudspeth, How hearing happens. *Neuron* **19**, 947–950 (1997).

4. A. Hudspeth, Integrating the active process of hair cells with cochlear function. *Nat. Rev. Neurosci.* **15**, 600–614 (2014).

5. A. Reinhardt-Rutland, S. Anstis, Auditory adaptation to gradual rise or fall in intensity of a tone. *Percept. Psychophys.* **31**, 63–67 (1982).

6. Z. Shu, N. Swindale, M. Cynader, Spectral motion produces an auditory after-effect. *Nature* **364**, 721–723 (1993).

7. B. C. Moore, Temporal integration and context effects in hearing. *J. Phon.* **31**, 563–574 (2003).

8. C. J. Dong, N. V. Swindale, P. Zakarauskas, V. Hayward, M. S. Cynader, The auditory motion after effect: Its tuning and specificity in the spatial and frequency domains. *Percept. Psychophys.* **62**, 1099–1111 (2000).

9. J. Blauert, *Spatial Hearing: The Psychophysics of Human Sound Localization* (MIT Press, 1997).

10. F. L. Wightman, D. J. Kistler, The dominant role of low-frequency interaural time differences in sound localization. *J. Acoust. Soc. Am.* **91**, 1648–1661 (1992).

11. E. A. Macpherson, J. C. Middlebrooks, Listener weighting of cues for lateral angle: The duplex theory of sound localization revisited. *J. Acoust. Soc. Am.* **111**, 2219–2236 (2002).

12. J. C. Middlebrooks, J. C. Makous, D. M. Green, Directional sensitivity of sound-pressure levels in the human ear canal. *J. Acoust. Soc. Am.* **86**, 89–108 (1989).

13. J. W. Strutt, On our perception of sound direction. *Philos. Mag.* **13**, 214–232 (1907).

14. D. W. Batteau, The role of the pinna in human localization. *Proc. R. Soc. Lond. B Biol. Sci.* **168**, 158–180 (1967).

15. S. Carlile, R. Martin, K. McAnally, Spectral information in sound localization. *Int. Rev. Neurobiol.* **70**, 399–434 (2005).

16. J. C. Middlebrooks, Sound localization. *Handb. Clin. Neurol.* **129**, 99–116 (2015).

17. P. Hofman, A. J. Van Opstal, Binaural weighting of pinna cues in human sound localization. *Exp. Brain Res.* **148**, 458–470 (2003).

18. S. Carlile, D. J. Pralong, The location-dependent nature of perceptually salient features of the human head-related transfer functions. *J. Acoust. Soc. Am.* **95**, 3445–3459 (1994).

19. N. Kopčo, B. G. Shinn-Cunningham, Effect of stimulus spectrum on distance perception for nearby sources. *J. Acoust. Soc. Am.* **130**, 1530–1541 (2011).

20. A. R. Cody, I. J. Russell, Outer hair cells in the mammalian cochlea and noise-induced hearing loss. *Nature* **315**, 662–665 (1985).

21. G. M. Clark, The multichannel cochlear implant for severe-to-profound hearing loss. *Nat. Med.* **19**, 1236–1239 (2013).

22. B. S. Wilson, Toward better representations of sound with cochlear implants. *Nat. Med.* **19**, 1245–1248 (2013).

23. D. Baby, A. Van Den Broucke, S. Verhulst, A convolutional neural-network model of human cochlear mechanics and filter tuning for real-time applications. *Nat. Mach. Intell.* **3**, 134–143 (2021).

24. D. R. Moore, R. V. Shannon, Beyond cochlear implants: Awakening the deafened brain. *Nat. Neurosci.* **12**, 686–691 (2009).

25. K. van der Heijden, J. P. Rauschecker, B. de Gelder, E. Formisano, Cortical mechanisms of spatial hearing. *Nat. Rev. Neurosci.* **20**, 609–623 (2019).

26. F. Denk, S. D. Ewert, B. Kollmeier, On the limitations of sound localization with hearing devices. *J. Acoust. Soc. Am.* **146**, 1732–1744 (2019).

27. R. Litovsky, A. Parkinson, J. Arcaroli, C. J. E. Sammeth, Simultaneous bilateral cochlear implantation in adults: A multicenter clinical study. *Ear Hear.* **27**, 714–731 (2006).

28. S. A. Ausili, B. Backus, M. J. Agterberg, A. J. van Opstal, M. M. van Wanrooij, Sound localization in real-time vocoded cochlear-implant simulations with normal-hearing listeners. *Trends Hear.* **23**, 2331216519847332 (2019).

29. H. A. Snapp, S. A. Ausili, Hearing with one ear: Consequences and treatments for profound unilateral hearing loss. *J. Clin. Med.* **9**, 1010 (2020).

30. A. Coudert, V. Gaveau, J. Gatel, G. Verdelet, R. Salemme, A. Farne, F. Pavani, E. Truy, Spatial hearing difficulties in reaching space in bilateral cochlear implant children improve with head movements. *Ear Hear.* **43**, 192–205 (2022).

31. J. Ikeya, A. Kawano, N. Nishiyama, S. Kawaguchi, A. Hagiwara, M. Suzuki, Long-term complications after cochlear implantation. *Auris Nasus Larynx* **40**, 525–529 (2013).

32. H. S. Lee, J. Chung, G. T. Hwang, C. K. Jeong, Y. Jung, J. H. Kwak, H. Kang, M. Byun, W. D. Kim, S. Hur, S. H. Oh, K. J. Lee, Flexible inorganic piezoelectric acoustic nanosensors for biomimetic artificial hair cells. *Adv. Funct. Mater.* **24**, 6914–6921 (2014).

33. Y. H. Jung, S. K. Hong, H. S. Wang, J. H. Han, T. X. Pham, H. Park, J. Kim, S. Kang, C. D. Yoo, K. J. Lee, Flexible piezoelectric acoustic sensors and machine learning for speech processing. *Adv. Mater.* **32**, e1904020 (2020).

34. G. Viola, J. Chang, T. Maltby, F. Steckler, M. Jomaa, J. Sun, J. Edusei, D. Zhang, A. Vilches, S. Gao, X. Liu, S. Saeed, H. Zabalawi, J. Gale, W. Song, Bioinspired multiresonant acoustic devices based on electrospun piezoelectric polymeric nanofibers. *ACS Appl. Mater. Interfaces* **12**, 34643–34657 (2020).

35. W. Yan, G. Noel, G. Loke, E. Meiklejohn, T. Khudiyev, J. Marion, G. Rui, J. Lin, J. Cherston, A. Sahasrabudhe, J. Wilbert, I. Wicaksono, R. W. Hoyt, A. Missakian, L. Zhu, C. Ma, J. Joannopoulos, Y. Fink, Single fibre enables acoustic fabrics via nanometre-scale vibrations. *Nature* **603**, 616–623 (2022).

36. H. S. Wang, S. K. Hong, J.H. Han, Y. H. Jung, H. K. Jeong, T. H. Im, C. K. Jeong, B.-Y. Lee, G. Kim, C. D. Yoo, K. J. Lee, Biomimetic and flexible piezoelectric mobile acoustic sensors with multiresonant ultrathin structures for machine learning biometrics. *Sci. Adv.* **7**, eabe5683 (2021).

37. S. Bacon, R. R. Fay, *Compression: From Cochlea to Cochlear Implants* (Springer, 2003).

38. G. von Békésy, Travelling waves as frequency analysers in the cochlea. *Nature* **225**, 1207–1209 (1970).

39. S. S. Narayan, A. N. Temchin, A. Recio, M. A. Ruggero, Frequency tuning of basilar membrane and auditory nerve fibers in the same cochleae. *Science* **282**, 1882–1884 (1998).

40. H. Shintaku, T. Nakagawa, D. Kitagawa, H. Tanujaya, S. Kawano, J. Ito, Development of piezoelectric acoustic sensor with frequency selectivity for artificial cochlea. *Sens. Actuators A Phys.* **158**, 183–192 (2010).

41. J. H. Han, J. H. Kwak, D. J. Joe, S. K. Hong, H. S. Wang, J. H. Park, S. Hur, K. J. Lee, Basilar membrane-inspired self-powered acoustic sensor enabled by highly sensitive multi tunable frequency band. *Nano Energy* **53**, 198–205 (2018).

42. L. H. Carney, Speeding up machine hearing. *Nat. Mach. Intell.* **3**, 190–191 (2021).

43. S. Haro, C. J. Smalt, G. A. Ciccarelli, T. F. Quatieri, Deep neural network model of hearing-impaired speech-in-noise perception. *Front. Neurosci.* **14**, 588448 (2020).

44. R. V. Shannon, F.-G. Zeng, V. Kamath, J. Wygonski, M. Ekelid, Speech recognition with primarily temporal cues. *Science* **270**, 303–304 (1995).

45. E. Formisano, M. De Martino, M. Bonte, R. Goebel, “Who” is saying “what”? Brain-based decoding of human voice and speech. *Science* **322**, 970–973 (2008).

46. D. Ward, D. MacKay, Fast hands-free writing by gaze direction. *Nature* **418**, 838–838 (2002).

47. H. Guo, X. Pu, J. Chen, Y. Meng, M. H. Yeh, G. Liu, Q. Tang, B. Chen, D. Liu, S. Qi, C. Wu, C. Hu, J. Wang, Z. L. Wang, A highly sensitive, self-powered triboelectric auditory sensor for social robotics and hearing aids. *Sci. Robot.* **3**, eaat2516 (2018).

48. N. Ma, J. A. Gonzalez, G. J. Brown, Robust binaural localization of a target sound source by combining spectral source models and deep neural networks. *IEEE/ACM Trans. Audio Speech Lang. Process.* **26**, 2122–2131 (2018).

49. W. Song, A smart sensor that can be woven into everyday life. *Nature* **603**, 585–586 (2022).

50. C. Lang, J. Fang, H. Shao, X. Ding, T. Lin, High-sensitivity acoustic sensors from nanofibre webs. *Nat. Commun.* **7**, 11108 (2016).

51. M. Toda, M. Thompson, Contact-type vibration sensors using curved clamped PVDF film. *IEEE Sens. J.* **6**, 1170–1177 (2006).

52. A. C. Cameron, F. A. G. Windmeijer, An R-squared measure of goodness of fit for some common nonlinear regression models. *J. Econom.* **77**, 329–342 (1997).

53. Z. Li, F. Liu, W. Yang, S. Peng, J. Zhou, A survey of convolutional neural networks: Analysis, applications, and prospects. *IEEE Trans. Neural Netw. Learn. Syst.* **33**, 6999–7019 (2021).

54. S. Xie, R. Girshick, P. Dollár, Z. Tu, K. He, “Aggregated residual transformations for deep neural networks,” in *2017 IEEE Conference on Computer Vission and Pattern Recognition (CVPR)* (IEEE, 2017), pp. 1492–1500.

55. Z. Niu, G. Zhong, H. J. N. Yu, A review on the attention mechanism of deep learning. *Neurocomputing* **452**, 48–62 (2021).

56. Z. Zhang, M. Sabuncu, "Generalized cross entropy loss for training deep neural networks with noisy labels," in *32nd Conference on Neural Information Processing System (NeurIPS 2018)* (Curran Associates Inc., 2018), pp. 8792–8802.

57. Q. Yang, P. Yan, Y. Zhang, H. Yu, Y. Shi, X. Mou, M. K. Kalra, Y. Zhang, L. Sun, G. Wang, Low-dose CT image denoising using a generative adversarial network with Wasserstein distance and perceptual loss. *IEEE Trans. Med. Imaging* **37**, 1348–1357 (2018).

58. S. Wang, T. Zhang, K. Li, S. Ma, M. Chen, P. Lu, L. Wei, Flexible piezoelectric fibers for acoustic sensing and positioning. *Adv. Electron. Mater.* **3**, 1600449 (2017).

59. Z. Lin, G. Zhang, X. Xiao, C. Au, Y. Zhou, C. Sun, Z. Zhou, R. Yan, E. Fan, S. Si, L. Weng, S. Mathur, J. Yang, J. Chen, A personalized acoustic interface for wearable human–machine interaction. *Adv. Funct. Mater.* **32**, 2109430 (2022).

60. M. Ha, S. Lim, J. Park, D. S. Um, Y. Lee, H. Ko, Bioinspired interlocked and hierarchical design of ZnO nanowire arrays for static and dynamic pressure-sensitive electronic skins. *Adv. Funct. Mater.* **25**, 2841–2849 (2015).

61. S. Kang, S. Cho, R. Shanker, H. Lee, J. Park, D. S. Um, Y. Lee, H. Ko, Transparent and conductive nanomembranes with orthogonal silver nanowire arrays for skin-attachable loudspeakers and microphones. *Sci. Adv.* **4**, eaas8772 (2018).

62. S. Lee, J. Kim, H. Roh, W. Kim, S. Chung, W. Moon, K. Cho, A high-fidelity skin-attachable acoustic sensor for realizing auditory electronic skin. *Adv. Mater.* **34**, e2109545 (2022).

63. Y. H. Jung, T. X. Pham, D. Issa, H. S. Wang, J. H. Lee, M. Chung, B. Y. Lee, G. Kim, C. D. Yoo, K. J. Lee, Deep learning-based noise robust flexible piezoelectric acoustic sensors for speech processing. *Nano Energy* **101**, 107610 (2022).

64. J. Jang, J. Lee, S. Woo, D. J. Sly, L. J. Campbell, J. H. Cho, S. J. O'Leary, M. H. Park, S. Han, J. W. Choi, J. Hun Jang, H. Choi, A microelectromechanical system artificial basilar membrane based on a piezoelectric cantilever array and its characterization using an animal model. *Sci. Rep.* **5**, 12447 (2015).

65. W. Wang, P. N. Stipp, K. Ouaras, S. Fathi, Y. Y. S. Huang, Broad bandwidth, self-powered acoustic sensor created by dynamic near-field electrospinning of suspended, transparent piezoelectric nanofiber mesh. *Small* **16**, e2000581 (2020).

66. M. O. G. Nayeem, S. Lee, H. Jin, N. Matsuhisa, H. Jinno, A. Miyamoto, T. Yokota, T. Someya, All-nanofiber–based, ultrasensitive, gas-permeable mechanoacoustic sensors for continuous long-term heart monitoring. *Proc. Natl. Acad. Sci. U.S.A.* **117**, 7063–7070 (2020).

67. D. Y. Park, D. J. Joe, D. H. Kim, H. Park, J. H. Han, C. K. Jeong, H. Park, J. G. Park, B. Joung, K. J. Lee, Self-powered real-time arterial pulse monitoring using ultrathin epidermal piezoelectric sensors. *Adv. Mater.* **29**, 1702308 (2017).

68. H. Shao, H. Wang, Y. Cao, X. Ding, J. Fang, W. Wang, X. Jin, L. Peng, D. Zhang, T. Lin, High-performance voice recognition based on piezoelectric polyacrylonitrile nanofibers. *Adv. Electron. Mater.* **7**, 2100206 (2021).

69. A. Sultana, M. M. Alam, S. K. Ghosh, T. R. Middya, D. Mandal, Energy harvesting and self-powered microphone application on multifunctional inorganic-organic hybrid nanogenerator. *Energy* **166**, 963–971 (2019).

70. X. Zhou, K. Parida, O. Halevi, Y. Liu, J. Xiong, S. Magdassi, P. S. Lee, All 3D-printed stretchable piezoelectric nanogenerator with non-protruding kirigami structure. *Nano Energy* **72**, 104676 (2020).

71. R. Peng, B. Zhang, G. Dong, Y. Wang, G. Yang, J. Zhang, B. Peng, Y. Zhao, M. Liu, Enhanced piezoelectric energy harvester by employing freestanding single-crystal $BaTiO_3$ Films in PVDF-TrFE based composites. *Adv. Funct. Mater.* **34**, 2316519 (2024).

72. S. Mirjalali, R. Bagherzadeh, A. Mahdavi Varposhti, M. Asadnia, S. Huang, W. Chang, S. Peng, C. H. Wang, S. Wu, Enhanced piezoelectricity of PVDF-TrFE nanofibers by intercalating with electrosprayed $BaTiO_3$. *ACS Appl. Mater. Interfaces* **15**, 41806–41816 (2023).

73. Y. Cho, J. Jeong, M. Choi, G. Baek, S. Park, H. Choi, S. Ahn, S. Cha, T. Kim, D. S. Kang, J. Bae, J. J. Park, $BaTiO_3$@ PVDF-TrFE nanocomposites with efficient orientation prepared via

phase separation nano-coating method for piezoelectric performance improvement and application to 3D-PENG. *Chem. Eng. J.* **427**, 131030 (2022).

74. P. Wang, Z. Chen, C. Cao, P. Zhou, Y. Qi, T. Zhang, K. Liang, A flexible magneto-electric sensor with enhanced performance through flower-shaped BTO fillers in P (VDF-TrFE) matrix. *Sens. Actuators A: Phys.* **373**, 115426 (2024).

75. K. Kim, D. Choi, S. Ji, F. B. Iniguez, Y. J. Song, S. S. Yoon, J. Kim, S. An Highly transparent and flexible all-nanofiber-based piezocomposite containing $BaTiO_3$-embedded P(VDF-TrFE) nanofibers for harvesting and monitoring human kinetic movements. *Adv. Fiber Mater.* **6**, 1369–1386 (2024).

76. C. C. Hong, S. Y. Huang, J. Shieh, S.-H. Chen, Enhanced piezoelectricity of nanoimprinted Sub-20 nm poly(vinylidene fluoride–trifluoroethylene) copolymer nanograss. *Macromolecules* **45**, 1580–1586 (2012).

77. J. H. Yang, T. Ryu, Y. Lansac, Y. H. Jang, B. H. Lee, Shear stress-induced enhancement of the piezoelectric properties of PVDF-TrFE thin films. *Org. Electron.* **28**, 67–72 (2016).

78. M. Maturi, L. Migliorini, S. M. Villa, T. Santaniello, N. Fernandez-Delgado, S. I. Molina, P. Milani, A. Sanz de León, M. C. Franchini, 3D-printing of highly piezoelectric barium titanate polymer nanocomposites with surface-modified nanoparticles at low loadings. *Adv. Funct. Mater.* **35**, 2407077 (2025).

79. Z. Zhou, H. Tang, H. A. Sodano, Vertically aligned arrays of $BaTiO_3$ nanowires. *ACS Appl. Mater. Interfaces* **5**, 11894–11899 (2013).
